# Honokiol‐Magnolol‐Baicalin Possesses Synergistic Anticancer Potential and Enhances the Efficacy of Anti‐PD‐1 Immunotherapy in Colorectal Cancer by Triggering GSDME‐Dependent Pyroptosis

**DOI:** 10.1002/advs.202417022

**Published:** 2025-02-14

**Authors:** Quan Gao, Qinsong Sheng, Zijing Yang, ZhiYu Zhu, Lin Li, Lihui Xu, Jing Xia, Yunhao Qiao, Jie Gu, Xiaolong Zhu, Tian Xie, Xinbing Sui

**Affiliations:** ^1^ School of Pharmacy Hangzhou Normal University Hangzhou Zhejiang 311121 China; ^2^ State Key Laboratory of Quality Research in Chinese Medicines Faculty of Chinese Medicine Macau University of Science and Technology Macau 999078 P. R. China; ^3^ Department of Colorectal Surgery The First Affiliated Hospital School of Medicine Zhejiang University Hangzhou Zhejiang 310058 China

**Keywords:** combination therapies, GSDME, Huangqin Houpo decoction, metastatic colorectal cancer, pyroptosis

## Abstract

Significant progress is made in the treatment of metastatic colorectal cancer (mCRC) patients, however, therapeutic options remain limited for patients with mCRC. In recent years, traditional Chinese medicine (TCM) has gained significant attention. Among these, Huangqin Houpo decoction has demonstrated efficacy in mCRC treatment. Despite its promise, the active ingredients and mechanisms underlying its anticancer effects remain unclear. Using integrative pharmacological approaches, six compounds are identified as the primary active ingredients in Huangqin Houpo decoction. Among them, honokiol (H), magnolol (M), and baicalin (B) are found to exhibit a synergistic anticancer effect on CRC. The HMB combination significantly outperforms mono‐ or bi‐agent treatments in reducing tumor growth. Furthermore, the anticancer efficacy of the HMB combination surpasses that of medium‐ and high‐dose Huangqin Houpo decoction and the FOLFOX regimen. Notably, HMB is comparable in efficacy to the FOLFIRI regimen. Most importantly, HMB is shown to enhance the sensitivity of CRC cells to anti‐PD‐1 immunotherapy in vivo. Mechanistic studies reveal that the HMB combination exerts its synergistic anticancer effects and enhances anti‐PD‐1 immunotherapy by inducing GSDME‐dependent pyroptosis. Our study will hopefully provide a potential therapeutic strategy for mCRC patients in the future.

[Correction added on 25 February 2025, after first online publication: FOLFOIRI is changed to FOLFIRI.]

## Graphical abstract

In this study, Honokiol (H)‐magnolol (M)‐baicalin (B) is identified as the primary active ingredients of Huangqin Houpo decoction. The HMB combination not only possesses potent synergistic anticancer activity, but also enhances the effectiveness of anti‐PD‐1 immunotherapy by inducing GSDME‐dependent pyroptosis in CRC cells.

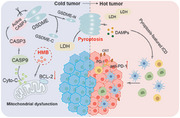

## 1 Introduction

Colorectal cancer (CRC) is one of the most prevalent and lethal gastrointestinal malignancies worldwide with high morbidity and mortality worldwide.^[^ ^1^ ^]^ Despite advancements in multimodal treatments – including surgery, chemotherapy, radiotherapy, molecular targeted therapies, and immunotherapy – CRC remains a significant clinical challenge.^[^ ^2^ ^]^ Although advancements in multimodal treatment have prolonged the survival of CRC patients after radical resection, ≈25% of patients present with metastatic CRC (mCRC).^[^ ^3^ ^]^ Current first‐line treatments for mCRC include bevacizumab or cetuximab in combination with 5‐fluorouracil (5‐FU)‐based chemotherapies, such as FOLFOX (5‐FU+oxaliplatin+calcium levofolinate) and FOLFIRI (5‐FU+irinotecan+calcium levofolinate).^[^ ^4^ ^]^ Additionally, immune checkpoint inhibitors have shown efficacy in mCRC patients with mismatch repair‐deficient (dMMR) or microsatellite instability‐high (MSI‐H) tumors.^[^ ^5^ ^]^ However, therapeutic options for mCRC patients are limited when the disease becomes refractory to these standard regimens. Therefore, new therapeutic strategies or more effective drugs for CRC patients are urgently needed.

Traditional Chinese medicine (TCM) has emerged as a promising source of novel anticancer drugs. Increasing evidence suggests that active ingredients from TCM formulas exhibit unique therapeutic effects in various diseases.^[^ ^6^ ^]^ For instance, the combination of active ingredients‐arsenic tetrasulfide, indirubin, and tanshinone IIA – from Compound Huangdai Tablets shows synergistic effects in both in vivo and in vitro models of acute promyelocytic leukemia (APL), surpassing the efficacy of Compound Huangdai Tablets.^[^ ^7^ ^]^ Similarly, active ingredients from Sini decoction (SND), including songorine, isoliquiritigenin, and 8‐gingerol, have been demonstrated to, in combination, mitigate dilated cardiomyopathy (DCM) by modulating mitochondrial energy metabolism and outperform the effect of any single ingredient.^[^ ^8^ ^]^ Huangqin Houpo decoction, a TCM formula comprising Huang Qin, Hou Po, Bai Shao, Zhi Ke, Chen Pi, Ge Gen, Chai Hu, and Gan Cao, has long been used to treat pyretic dysentery. Clinical practice has also suggested its efficacy in mCRC treatment. However, the active ingredients of this decoction and their possible underlying anticancer mechanisms remain largely unexplored.

Pyroptosis is a form of inflammatory programmed necrotic cell death mediated by the gasdermins family and the inflammatory caspases family. This process leads to membrane rupture and the release of cellular contents, triggering a robust immune response.^[^ ^9^ ^]^ Recent studies have highlighted pyroptosis as a promising strategy for inducing cancer cell death and inhibiting tumor proliferation and invasion.^[^ ^10^ ^]^ Additionally, pyroptosis is recognized as a form of immunogenic cell death (ICD), capable of enhancing tumor immunogenicity and promoting antitumor immunity. The induction of pyroptosis in tumors can convert cold tumors into hot tumors, thereby activating anti‐tumor immunity. Consequently, pyroptosis inducers have been investigated as sensitizers in combination with anti‐PD‐1 immunotherapy to suppress tumor growth.^[^ ^11^ ^]^ Currently, active ingredients derived from TCM have demonstrated the ability to induce pyroptosis in cancer cells.^[^ ^12^ ^]^ However, no studies to date have explored the relationship between pyroptosis and the combined use of active ingredients from Huangqin Houpo decoction.

In this study, we reported the development of a novel combination therapy consisting of honokiol (H), magnolol (M), and baicalin (B), which exhibits synergistic anticancer effects against CRC. Our results demonstrated that the HMB combination outperformed medium‐ and high‐dose Huangqin Houpo decoction, FOLFOX regimen, and was not inferior than that of the FOLFIRI regimen in animal experiments. Notably, the HMB combination significantly enhanced the therapeutic efficacy of anti‐PD‐1 immunotherapy, even achieving complete tumor elimination in an orthotopic MC38 CRC model. Mechanistic studies revealed that HMB exerted its synergistic anticancer effects by triggering GSDME‐dependent pyroptosis in CRC cells, organoids and various mouse tumor models. Importantly, the treatment showed no notable toxicity. Overall, our findings demonstrate that the HMB combination not only possesses potent synergistic anticancer activity, but also enhances the effectiveness of anti‐PD‐1 immunotherapy. This combination represents a promising candidate for CRC treatment, offering a potential therapeutic strategy to address unmet clinical needs.

## 2 Results

### 2.1 Characterization of the Chemical Compound Profile of Huangqin Houpo Decoction

**Figure 1:**
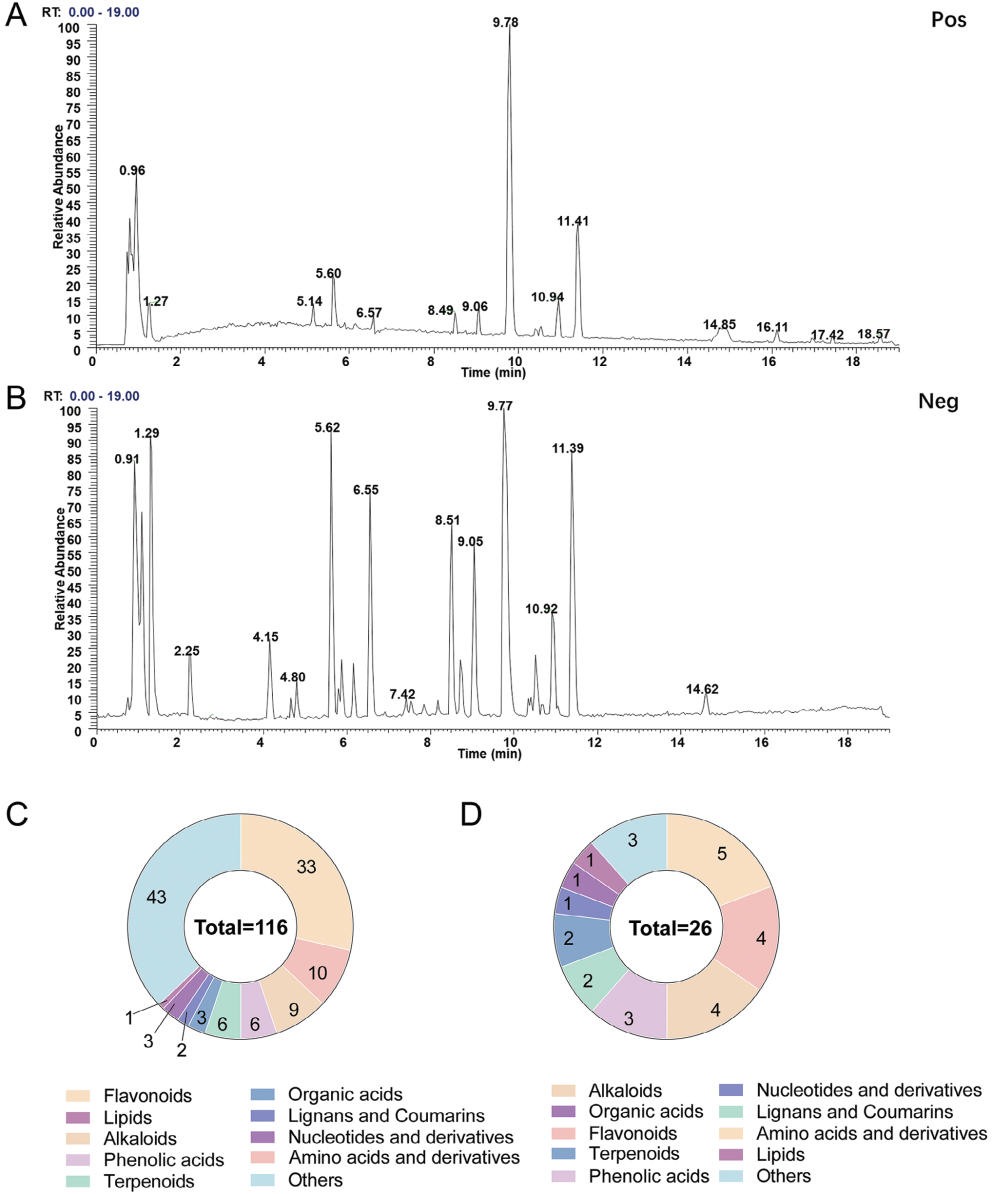
Chemical identification of Huangqin Houpo decoction. A) The chemical base peak ion chromatogram of Huangqin Houpo decoction in the positive ion mode. B) The BIP chromatogram of Huangqin Houpo decoction in the negative ion mode. C) Structural classification of compounds contained in the Huangqin Houpo decoction. D) Structural classification of prototype compounds in the serum.

Huangqin Houpo decoction is a classical TCM prescription, and its precise chemical characterization is essential for identifying active substances with anticancer properties. To achieve this, ultrahigh‐performance liquid chromatography coupled with Orbitrap high‐resolution mass spectrometry (UPLC‐Q‐Orbitrap MS) was employed to analyze the decoction's chemical profile. As shown in **Figure** **1**A,B, the primary chromatograms of Huangqin Houpo decoction were generated in both positive and negative ion modes. Chemical composition analysis was conducted using the Compound Discover software, with compounds cross‐referenced against the HMDB (https://hmdb.ca/) and PubChem (https://pubchem.ncbi.nlm.nih.gov/) databases. Elemental compositions were analyzed using Xcalibur software. Thirty‐one standard compounds (Dataset S1, Supporting Information) were used to enhance the accuracy and reliability of this characterization. Ultimately, a total of 116 high‐content chemical compounds of Huangqin Houpo decoction were detected, including 33 flavonoids, ten amino acids and derivatives, nine alkaloids, six phenolic acids, six terpenoids, three organic acids, two lignans and coumarins, three nucleotides and derivatives, one lipid, and 43 others (Figure 1C). Details about the retention times (RT), mass‐to‐charge ratios (m/z), mass errors, and molecular formulas of the detected compounds are provided in Dataset S1 (Supporting Information).

To investigate the bioavailability of these compounds, serum samples were analyzed after intragastric administration of Huangqin Houpo decoction using the UPLC‐MS/MS system (Figure S1A–D, Supporting Information). A total of 26 prototype compounds – those present in both the Huangqin Houpo decoction and serum were detected, including four flavonoids, four amino acids and derivatives, five alkaloids, three phenolic acids, two lignans and coumarins, two terpenoids, one nucleotide and derivative, one organic acid, one lipid, and three others (Figure 1D). Figure 1D summarizes these findings. Among the prototype compounds, 11 were commercially available as potential drug candidates for CRC treatment (Figure S2, Supporting Information). Their structures are shown in Figure S2 (Supporting Information). Dataset S2 (Supporting Information) provided detailed data, including RT, molecular weight (Da), formula, fragment ions, and compound identification.

### 2.2 Active Ingredients of Huangqin Houpo Decoction for CRC Treatment Are Determined

**Figure 2:**
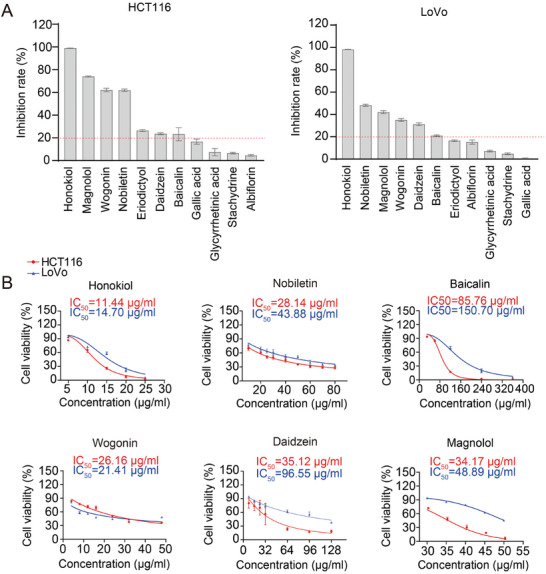
Screening of anti‐colorectal cancer compounds from Huangqin Houpo decoction. A) Preliminary screening of prototype compounds exhibiting anti‐colorectal cancer activity in HCT116 and LoVo cell lines. B) HCT116 and LoVo cell viability was evaluated using the CCK‐8 assay after 24‐h treatment with different concentrations of honokiol (H), nobiletin (N), baicalin (B), wogonin (W), daidzein (D), and magnolol (M). Data are represented as the mean ± S.D.

To identify active ingredients in Huangqin Houpo decoction with potential anti‐CRC effects, we evaluated 11 prototype compounds (marked with an asterisk in Figure S2, Supporting Information) in HCT116 and LoVo CRC cell lines. Our findings revealed that six compounds – honokiol, nobiletin, magnolol, wogonin, daidzein, and baicalin – exhibited significant anti‐CRC activity in both HCT116 and LoVo cell lines (**Figure** **2**A). The half‐maximal inhibitory concentration (IC_50_) values for these compounds were determined using the CCK‐8 assay (Figure 2B).

**Figure 3:**
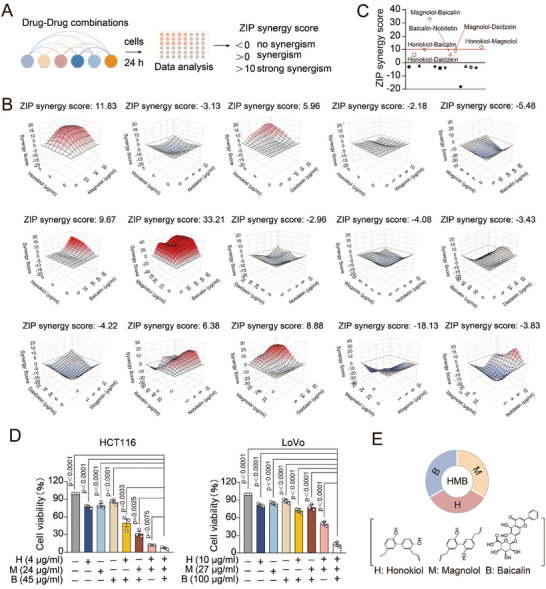
Evaluation of drug synergies. A) Study design. HCT116 cells were treated with two compounds for drug sensitivity. B,C) The synergistic effects were quantified using the ZIP synergy scores (ZIP Synergy scores greater than 0 were considered indicative of synergism, and scores above 10 were considered strongly synergistic) via the SynergyFinder software, based on the cell viability from different drug combinations. D) HCT116 (H: 4 µg mL⁻^1^; M: 24 µg mL⁻^1^; B: 45 µg mL⁻^1^) and LoVo (H: 10 µg mL⁻^1^; M: 27 µg mL⁻^1^; B: 100 µg mL⁻^1^) cell lines treated with H alone, M alone, B alone, H‐M, H‐B, M‐B or the combination of H, M, B. HCT116 (H: 4 µg mL⁻^1^; M: 24 µg mL⁻^1^; B: 45 µg mL⁻^1^) CI value: 0.215; LoVo (H: 10 µg mL⁻^1^; M: 27 µg mL⁻^1^; B: 100 µg mL⁻^1^) CI value: 0.236. CI value indicates a synergistic effect. 0.9 ≤ CI < 1.1 indicates superposition effect, 0.8 ≤ CI < 0.9 indicates low synergy effect, 0.6 ≤ CI < 0.8 indicates moderate synergy effect, 0.4 ≤ CI < 0.6 indicates high synergy effect, 0.2 ≤ CI < 0.4 indicates strong synergy effect. E) The HMB combination was considered as a combinatorial drug with anti‐colorectal cancer potential.

To further narrow down the active ingredient combinations, we conducted a series of experiments. First, we assessed the synergistic effects of two‐drug combinations in HCT116 cell lines. Using various concentrations of these combinations, we measured cell viability by the CCK‐8 assay and quantified the synergistic effects by ZIP synergy scores. A ZIP Synergy score >0 indicated synergy, while a score >10 denoted strong synergy, as calculated by SynergyFinder software^[^ ^13^ ^]^ (**Figure** **3**A). Notably, combinations such as honokiol‐magnolol (ZIP: 11.83), honokiol‐baicalin (ZIP: 9.67), honokiol‐daidzein (ZIP: 5.96), magnolol‐baicalin (ZIP: 33.21), magnolol‐daidzein (ZIP: 8.88), and baicalin‐nobiletin (ZIP: 6.38) exhibited synergistic inhibition in HCT116 cells (Figure 3B,C). Intriguingly, a strong synergistic effect was observed among the three drugs: honokiol‐magnolol‐baicalin and honokiol‐magnolol‐daidzein. According to the ZIP scores, honokiol (H)‐magnolol (M)‐baicalin (B) has a better synergistic effect than honokiol (H)‐magnolol (M)‐daidzein (D). So, the honokiol‐magnolol‐baicalin (HMB) was selected for subsequent study. To further evaluate the synergistic potential of H, M, and B, we selected the low concentrations of these compounds that exhibited synergistic effects in pairwise combinations for cell viability assays. Using the Compusyn software,^[^ ^14^ ^]^ the combination index (CI) of H‐M‐B was obtained, when CI values below 0.4 indicate strong synergy between the three drugs. The CI values for HMB treatment were 0.215 in HCT116 cells and 0.236 in LoVo cells (HCT116: H 4 µg mL⁻^1^, M 24 µg mL⁻^1^, B 45 µg mL⁻^1^; LoVo: H 10 µg mL⁻^1^, M 27 µg mL⁻^1^, B 100 µg mL⁻^1^) (Figure S3A, Supporting Information), indicating a robust synergistic activity. Moreover, the anticancer effects of the HMB combination were significantly higher than those of single‐ or dual‐agent treatments, with an inhibition rate exceeding 90% (Figure 3D). Therefore, we believe that the concentrations in this ratio have a strong synergistic effect and can be defined as the optimal synergistic ratio. In light of these findings, the HMB combination is considered as a combinatorial drug with anti‐colorectal cancer potential (Figure 3E).

### 2.3 HMB Synergistically Inhibits Cell Proliferation and Triggers Cell Death in CRC Cells and Organoid Models

**Figure 4:**
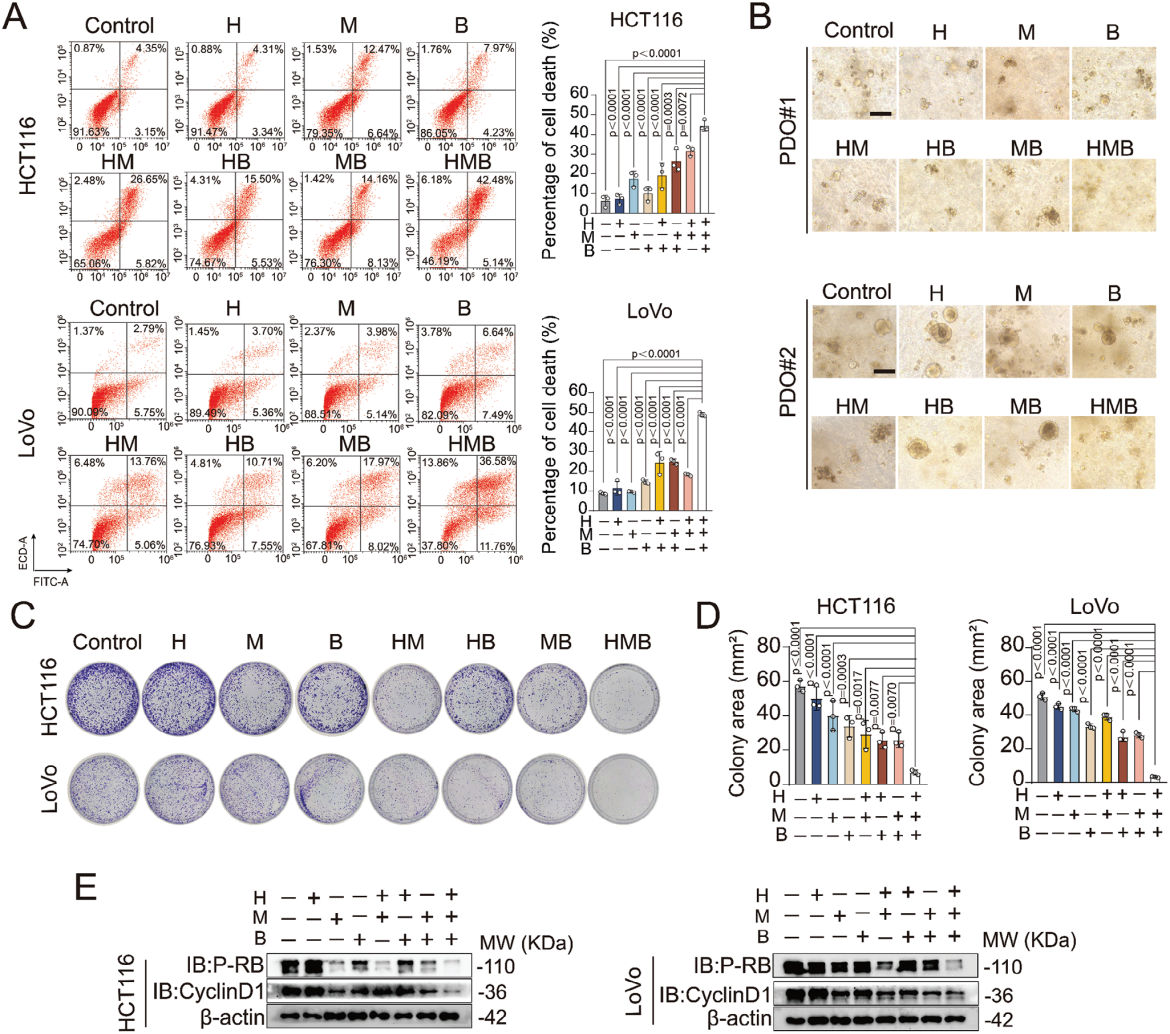
HMB synergistically inhibits cell proliferation and triggers cell death in CRC cells and organoid models. A) Cell death rate was assessed by flow cytometry after staining with phartmingen Annexin V‐FITC Apoptosis Detection Kit in HCT116 and LoVo cells following H, M and B treatment for 24 h. B) PDO#1 and PDO#2 were derived from the patient's colorectal cancer tissue composition. Morphological changes of organoids from two patients with colorectal cancer treated with honokiol‐magnolol‐baicalin for 48 h were observed, Scale bar = 200 µm. C,D) Colony‐forming of honokiol‐magnolol‐baicalin treatment. HCT116 and LoVo cells were treated with drugs for 2 weeks. Colony formation was fixed in methanol, stained with crystal violet, and counted. The mean ± SD is presented, n = 3. E) Western blotting analysis of p‐RB, CyclinD1 expression in control, H, M, B, HM, HB, MB, and HMB treatment groups.

To assess the effects of the HMB combination on CRC cells, we observed morphological changes under a microscope. HMB treatment reduced cell proliferation and increased the number of floating, non‐adherent cells in the culture medium (Figure S3B, Supporting Information), suggesting a combination of antiproliferative and cell death‐inducing effects. Flow cytometry analysis with Annexin V/PI dual staining confirmed a significant increase in cell death in HCT116 and LoVo cells following treatment with the optimal HMB concentration (**Figure** **4**A). To validate these findings in patient‐derived models, organoids generated from CRC samples were treated with HMB. Microscopic observations revealed that HMB inhibited organoids growth and disrupted their cluster‐like morphology (Figure 4B).

To further evaluate antiproliferative effects, colony formation assays were performed. HMB treatment significantly reduced colony formation compared with single or dual‐agent treatments (Figure 4C,D). Cell proliferation is closely associated with cell cycle regulation characterized primarily by the disruption of cell cycle regulatory mechanisms.^[^ ^15^ ^]^ Dysregulation of cyclin‐dependent kinases (CDKs) and cyclins, key proteins regulating the cell cycle, is closely associated with the unchecked proliferation of cancer cells.^[^ ^16^ ^]^ To examine the impact of the HMB combination on these proteins, western blot analysis was performed. According to western blotting results, HMB treatment induced G_0_/G_1_ phase arrest, accompanied by reduced expression of cyclin D1 and p‐RB expression in both HCT116 and LoVo cells. These effects were more pronounced with HMB than with mono‐ or bi‐agents treatments (Figure 4E). These results suggest that H, M, and B may exert a synergistic anti‐proliferative effect by promoting G_0_/G_1_ arrest in CRC cells.

To examine the impact of HMB on cell migration, wound healing was performed. As shown in Figure S4 (Supporting Information), co‐treatment with HMB significantly reduced cell migration compared with mono‐ or bi‐agents treatment.

These comprehensive findings demonstrate that the HMB combination exerts synergistic anti‐CRC effects by inhibiting cell proliferation, inducing cell death, arresting the cell cycle, and reducing cell migration in vitro, compared with either mono‐ or bi‐agent treatments.

### 2.4 HMB Synergistically Induces Pyroptosis and Apoptosis

**Figure 5:**
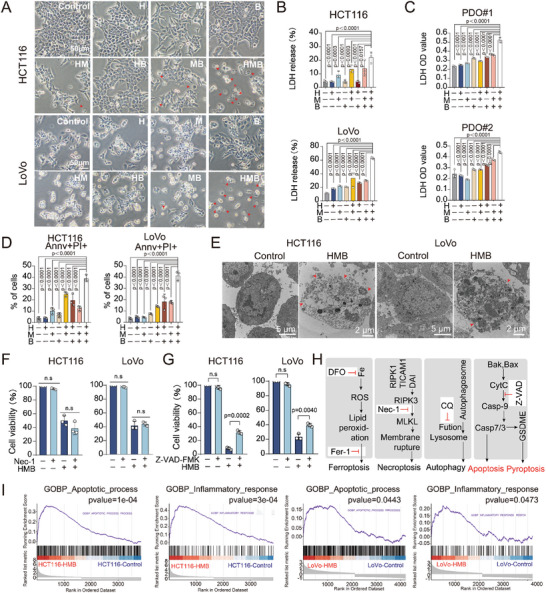
HMB synergistically induces pyroptosis and apoptosis. A,B) HCT116 and LoVo cell lines were treated with H, M, and B treatment. 24 h later, A) Cell morphological changes and B) LDH release were measured. Scale bar = 50 µm. The mean ± SD is shown, n = 3. C) LDH release of organoids constructed from two patients with colorectal cancer treated with honokiol‐magnolol‐baicalin for 48 h were measured. The mean ± SD is shown, n = 3. D) Annexin V‐FITC+PI+ were assessed by flow cytometry after staining with phartmingen Annexin V‐FITC Apoptosis Detection Kit in HCT116 and LoVo cells following H, M, and B treatment for 24 h. The mean ± SD is shown, n = 3. E) Cell membrane morphology was observed by transmission electron microscopy (red arrowheads: arising pore from the plasma membrane). F,G) Cell viability of CRC cells was detected after the treatment with the HMB in combination with different cell death inhibitors for 24h, including pan caspase inhibitor Z‐VAD‐FMK (z‐VAD, 10µM) and necroptosis inhibitor necrostatin‐1 (Nec‐1, 10µM). The mean ± SD is shown, n = 3. H) The key regulatory molecules of different cell death pathways, represented by arrows. I) GSEA suggested that the associated inflammatory pathways and apoptotic process were activated after the HMB combination treatment.

In this study, we observed that treatment with the HMB combination significantly induced cell death, characterized by distinct morphological changes such as cell swelling and extensive plasma membrane blebbing (**Figure** **5**A). These features differ from the classic hallmarks of apoptosis and instead align with the morphological characteristics of pyroptosis. Supporting this observation, lactate dehydrogenase (LDH) release assay revealed a marked increase in LDH levels in the HMB combination‐treated group, surpassing those observed in the mono‐ or bi‐agents groups in both CRC cells and organoids models. This confirms plasma membrane rupture, a key feature of pyroptosis (Figure 5B,C). Additionally, Annv/PI dual staining assays demonstrated a significant rise in the population of Annv/PI double‐positive cells upon HMB treatment (Figure 5D). Transmission electron microscopy further corroborated these findings, displaying similar morphological changes consistent with pyroptosis (Figure 5E). Given that necroptosis also features membrane rupture, cell swelling, and lysis,^[^ ^17^ ^]^ the necroptosis inhibitor necrostatin‐1 (Nec‐1) was employed to evaluate its involvement. Nec‐1 failed to prevent cell membrane rupture, LDH release, or cell death, suggesting that necroptosis was not induced by the HMB combination treatment (Figures 5F and S5A, Supporting Information). To explore the involvement of other cell death mechanisms, various inhibitors targeting different cell death programs, including autophagy, ferroptosis, and pan caspase inhibitors, were used. Notably, the pan caspase inhibitor Z‐VAD‐FMK effectively rescued the cell death (Figures 5G and S5B, Supporting Information), implying that the HMB combination triggered caspase‐dependent cell death mechanisms such as apoptosis or pyroptosis (Figure 5H).^[^ ^12^, ^18^ ^]^ Gene set enrichment analysis (GSEA) further revealed that the HMB combination activated pathways related to both the apoptotic process and the inflammatory response (Figure 5I). This is consistent with the notion that pyroptosis is an inflammatory form of cell death.^[^ ^19^ ^]^ Collectively, these findings suggest that the HMB combination induces both apoptosis and pyroptosis in CRC cells.

### 2.5 HMB Synergistically Induces Pyroptosis by Promoting the Cleavage of GSDME

**Figure 6:**
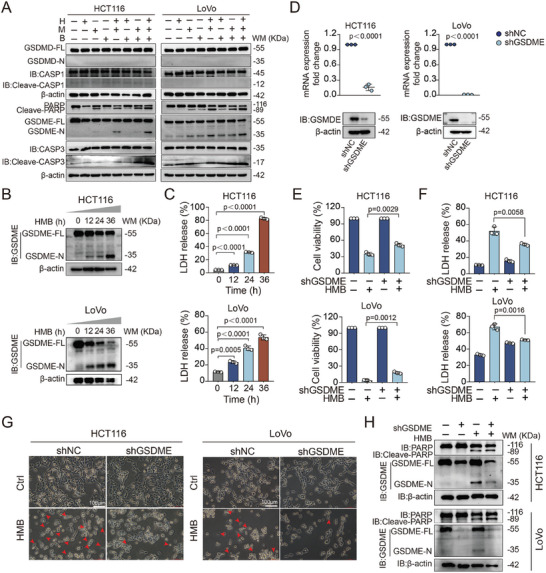
The combination of HMB synergistically induces pyroptosis by promoting the cleavage of GSDME. A) Western blotting analysis of GSDMD‐FL, GSDMD‐N, Caspase 1, Cleave‐caspase 1, GSDME‐FL, GSDME‐N, PARP, Caspase 3 and Cleave‐caspase 3 expression by H, M, and B treatment. B,C) The levels of GSDME‐N proteins and LDH release were assessed in HCT116 and LoVo cell lines at 12h, 24h, and 36h time points after the HMB treatment. The mean ± SD is shown, n = 3. D) GSDME knock down levels were verified by RT‐PCR and Western blotting. E) CCK8 analysis was performed to investigate the cell viability of HCT116 and LoVo subjected to stable knockdown of GSDME with the indicated doses of HMB treatment for 24 h. The mean ± SD is shown, n = 3. F) LDH release were measured under the HMB treatment. The mean ± SD is shown, n = 3. G) The typical pyroptosis morphology following H, M, and B co‐treatment for 24 h in GSDME knockdown cells, scale bar = 100 µm. H) Western blotting analysis of GSDME‐FL, GSDME‐N, PARP and Cleave‐PARP expression by H, M, and B treatment in GSDME knockdown cells.

Pyroptosis is a well‐characterized inflammatory form of programmed cell death, primarily mediated by gasdermin proteins.^[^ ^20^ ^]^ To confirm pyroptosis induction, western blotting analyses were conducted to detect gasdermin‐related proteins, including GSDME and GSDMD, in HCT116 and LoVo cells treated with the HMB combination. The results showed that the HMB treatment activated GSDME, but not GSDMD (**Figure** **6**A). To determine whether pyroptosis was mediated through the caspase‐1/GSDMD‐dependent or caspase‐3/GSDME‐dependent pathway, we analyzed their expression levels through western blotting. HMB treatment specifically induced the cleavage of caspase‐3/GSDME but not caspase‐1/GSDMD, indicating that pyroptosis occurred through the caspase‐3/GSDME axis (Figure 6A). In agreement with this result, GSDME cleavage and LDH release were increased in a time‐dependent manner upon exposure to HMB treatment (Figure 6B,C).

To confirm the role of GSDME in HMB‐induced pyroptosis, GSDME was knocked down using shRNA in HCT116 and LoVo cells (Figure 6D). As expected, GSDME knockdown reduced the cytotoxicity effects of HMB treatment, attenuating pyroptosis‐associated morphological changes and LDH release (Figure 6E–G). Interestingly, GSDME knockdown shifted some cells toward apoptosis, further underscoring the pivotal role of GSDME in HMB‐induced pyroptosis (Figure 5H). Therefore, GSDME is the target for the synergistic anti‐cancer effects of HMB.

**Figure 7:**
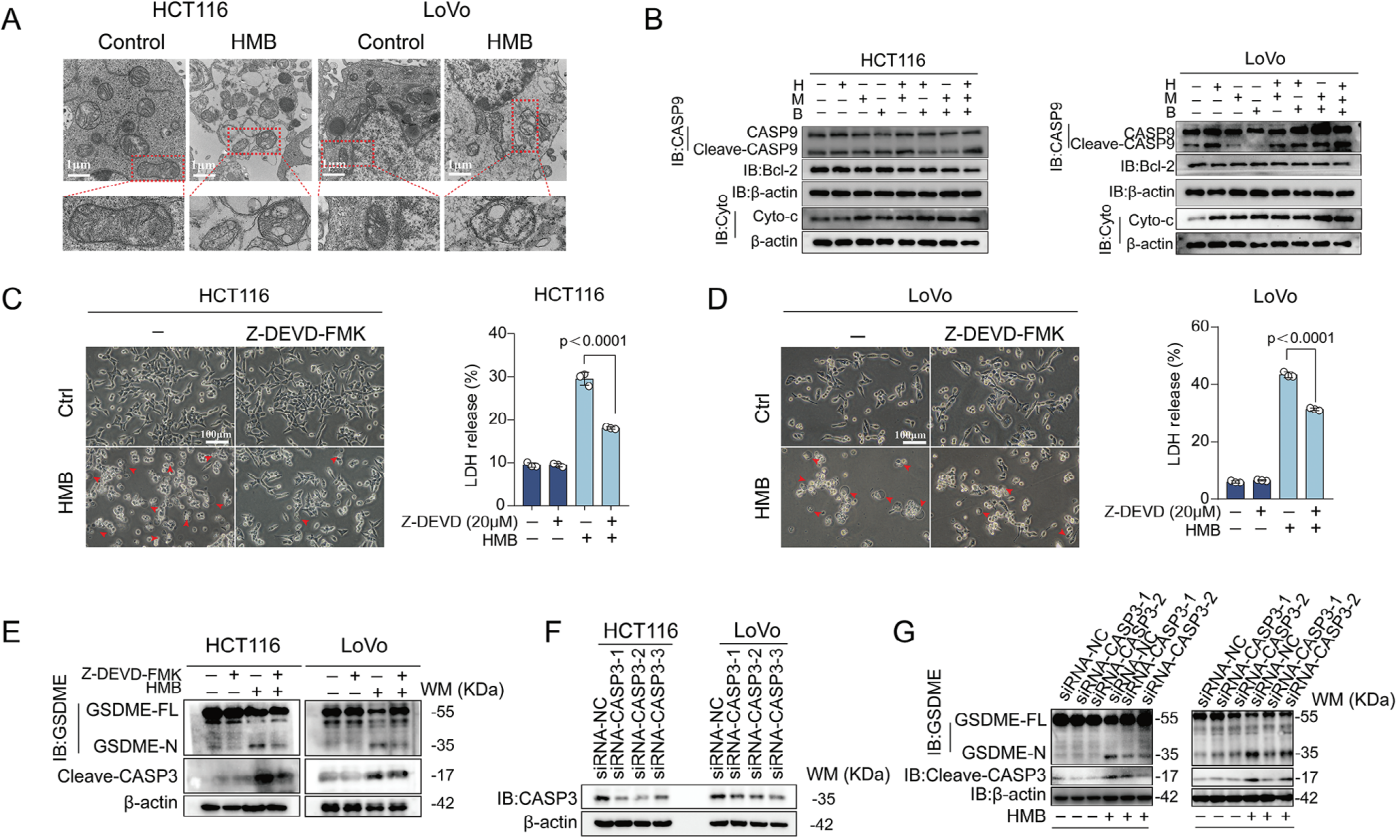
The HMB combination activated the mitochondrial intrinsic apoptotic pathway to elicit GSDME‐dependent pyroptosis. A) Mitochondrial morphology was observed by transmission electron microscopy B) The protein expression was detected by Western blotting. C,D) The typical pyroptosis morphology and LDH release were detected after the combination of caspase‐3 inhibitor Z‐DEVD‐FMK and the HMB, scale bar = 100 µm. The mean ± SD is shown, n = 3. E) HCT116 and LoVo cells were tested under the HMB and Z‐DEVD‐FMK combination treatment for activation of GSDME. F,G) HCT116 and LoVo cells of caspase‐3 knockdown were tested under the HMB treatment for activation of GSDME.

The intrinsic mitochondrial apoptotic pathway is a key regulator of cell death.^[^ ^21^ ^]^ Specifically, intracellular factors, such as cytochrome C, released from mitochondria are known to activate caspase‐3 and subsequently induce GSDME‐dependent pyroptosis.^[^ ^22^ ^]^ Therefore, mitochondrial changes and damage are key indicators of this process.^[^ ^23^ ^]^ To investigate whether mitochondrial dysfunction contributed to HMB‐induced pyroptosis, transmission electron microscopy confirmed these observations, showing swollen mitochondria with fragmented and blurred cristae in HCT116 and LoVo cells (**Figure** **7**A). Western blot analysis demonstrated that HMB treatment reduced Bcl‐2 expression, promoted cytochrome c release from mitochondria, and activated caspase‐9 (Figure 7B).

Caspase‐3 activation, a downstream event in this pathway, led to GSDME cleavage and pyroptosis.^[^ ^22b^ ^]^ The addition of the caspase‐3 inhibitor Z‐DEVD‐FMK mitigated pyroptosis‐associated morphological changes, LDH release, and GSDME cleavage in HCT116 and LoVo cells (Figure 7C–E). Similarly, caspase‐3 knockdown using siRNAs decreased GSDME cleavage, confirming the crucial role of caspase‐3 in HMB‐induced pyroptosis (Figure 7F,G). In summary, these results collectively reveal that the HMB combination induces robust GSDME‐dependent pyroptosis through the intrinsic mitochondrial apoptotic pathway.

### 2.6 In Vivo Antitumor Effects of the HMB Combination

**Figure 8:**
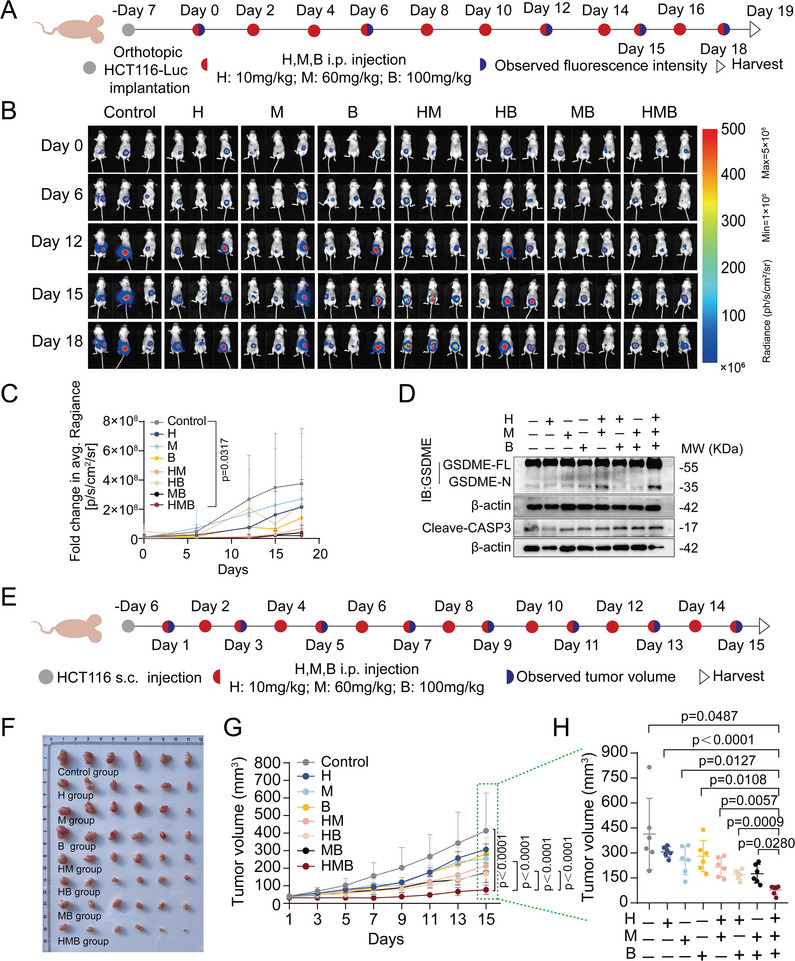
In vivo antitumor effects of the HMB combination. A) The experimental design involved orthotopic HCT116‐Luc (s.c.) tumor inoculation and treatment scheduling in athymic nude mice. Seven day after tumor inoculation, the mice were treated with H (10 mg kg⁻^1^), M (60 mg kg⁻^1^), and B (100 mg kg⁻^1^) every 2 days. B) Orthotopic HCT116‐Luc CRC tumor growth was monitored by evaluating the average radiance within the tumor sites by bioluminescence imaging on days 0, 6, 12, 15, 18. C) Average radiance of tumor burden (fold changes) calculated through bioluminescence imaging at different time points. Data shown as means ± S.D. (n = 3). D) The expression of GSDME and cleave‐caspase3 in the tumor tissues of different treatment groups. E) The experimental design involved subcutaneous (s.c.) tumor inoculation and treatment scheduling in HCT116 tumor‐bearing athymic nude mice. Six day after tumor inoculation, the mice were treated daily with H 10 mg kg⁻^1^, M 60 mg kg⁻^1^, and B 100 mg kg⁻^1^. F) Photographs of excised tumors from HCT116 xenograft‐bearing mice across treatment groups were taken on day 15 (n = 6). G) Tumor growth curves for all treatment groups, presented as the mean ± S.D (n = 6). H) Tumor volumes at the experimental endpoint (day 13) for all groups, presented as the mean ± SD (n = 6).

We initially assessed the effect of HMB treatment on MC38 colorectal cancer cells in vitro and observed results consistent with those seen in HCT116 and LoVo cells. HMB treatment caused cell membrane rupture and increased LDH release in MC38 cells (Figure S6A,B, Supporting Information). To evaluate the synergistic therapeutic potential of the HMB combination in vivo, a subcutaneous MC38 tumor model was established. Two dosages of the HMB combination were administered daily: low‐dose (H 10 mg kg⁻^1^, M 60 mg kg⁻^1^, B 100 mg kg⁻^1^) and medium‐dose (H 15 mg kg⁻^1^, M 90 mg kg⁻^1^, B 150 mg kg⁻^1^) (Figure S6C, Supporting Information). Both dosages exhibited significantly superior efficacy compared with mono‐ or bi‐agent treatments, demonstrating the synergistic anticancer effects of the HMB combination in vivo (Figure S6D, Supporting Information). Consequently, the low‐dose HMB combination was selected for subsequent experiments. To further evaluate its therapeutic potential, an orthotopic CRC model was established by injecting luciferase‐expressing HCT116‐Luc cells into the colonic mesentery of immunodeficient nude mice. Seven days post‐inoculation, the mice were randomized into eight groups, and treated via intraperitoneal injection every other day for 18 days (**Figure** **8**A). Mice treated with the HMB combination exhibited significant reductions in fluorescence intensity compared with control groups (Figure 8B,C). Consistently, in vivo activation of caspase‐3 and GSDME was detected in the HMB‐treated group, aligning with the in vitro findings (Figure 8D). A subcutaneous HCT116 tumor model was also used to validate the efficacy of the HMB combination. Various drug combinations were administered daily via intraperitoneal injection (Figure 8E). Mice treated with saline exhibited rapid tumor growth, while H, M, B, and their pairwise combinations demonstrated moderate antitumor activity. Importantly, the HMB combination displayed the most robust anticancer effects compared with mono‐ or bi‐agent treatments (Figure 8F–H). H&E staining and blood biochemical analysis revealed no significant toxic effects on major organs, including the heart, liver, spleen, lungs, and kidneys (Figure S7A, Supporting Information). Serum biochemical parameters remained within normal ranges (Figure S7B, Supporting Information), confirming the safety of the HMB therapy. Collectively, these results demonstrate that the HMB combination synergistically inhibited tumor growth and induced pyroptosis in vivo.

**Figure 9:**
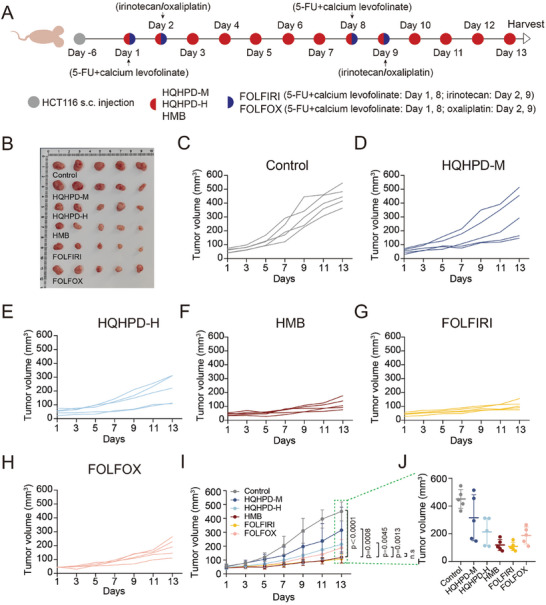
Comparison among the HMB combination, Huangqin Houpo Decoction, FOLFOX, and FOLFIRI. A) The experimental design involved subcutaneous (s.c.) tumor inoculation and treatment scheduling in HCT116 tumor‐bearing athymic nude mice. After 6 days of tumor inoculation, mice were treated daily with honokiol‐magnolol‐baicalin (HMB), Huangqin Houpo decoction‐medium dose (HQHPD‐M), Huangqin Houpo decoction‐high dose (HQHPD‐H), FOLFOX regimen (5‐fluorouracil (5‐FU), oxaliplatin, calcium levofolinate), and FOLFIRI regimen (5‐FU, irinotecan, calcium levofolinate).The FOLFOX group received 5‐FU (22.5 mg kg⁻^1^) and calcium levofolinate (33.75 mg kg⁻^1^) on days 1 and 8, with oxaliplatin (9.375 mg kg⁻^1^) on days 2 and 9. The FOLFIRI group received 5‐FU (22.5 mg kg⁻^1^) and calcium levofolinate (33.75 mg kg⁻^1^) on days 1 and 8, with irinotecan (15 mg kg⁻^1^) on days 2 and 9. The HMB group was treated with H (10 mg kg⁻^1^), M (60 mg kg⁻^1^), and B (100 mg kg⁻^1^) daily, while the HQHPD‐M group received 15.21 g kg⁻^1^, and the HQHPD‐H group received 30.42 g kg⁻^1^ of the decoction daily. B) Photographs of the excised tumors from HCT116 xenograft‐bearing mice across different treatment groups were captured on day 13 (n = 5). C‐H) Individual tumor growth kinetics in control (C), HQHPD‐M (D), HQHPD‐H (E), HMB (F), FOLFIRI (G) and FOLFOX (H) (n = 5). I) Tumor growth curve for all treatment groups. Data are presented as the mean ± S.D (n = 5). J) tumor volumes at the experimental endpoint (day 13) in all groups. Data was shown as mean ± S.D (n = 5).

Currently, standard therapies for mCRC, such as bevacizumab or cetuximab combined with 5‐FU‐based chemotherapy, including FOLFOX and FOLFIRI, offer limited options once the disease becomes refractory to these standard regimens. To evaluate the clinical potential of the HMB combination, a subcutaneous xenograft model was used to compare its efficacy against medium‐ and high‐dose Huangqin Houpo decoction group, the FOLFOX regimen group, and the FOLFIRI regimen group (**Figure** **9**A). Tumor inhibition in the HMB group was superior to the medium‐ and high‐dose Huangqin Houpo decoction groups, and its anticancer efficacy exceeded that of FOLFOX and was comparable to FOLFIRI (Figure 9B–J). H&E staining and blood biochemical analyses across various organs showed no significant toxicity (Figure S8, Supporting Information). Overall, these findings suggest that the HMB combination, as the principal anticancer components of Huangqin Houpo decoction, holds significant clinical translational potential and may offer a promising therapeutic option for recurrent CRC patients.

### 2.7 HMB Combination Enhances the Sensitivity of CRC Tumors to Anti‐PD‐1 Treatment

**Figure 10:**
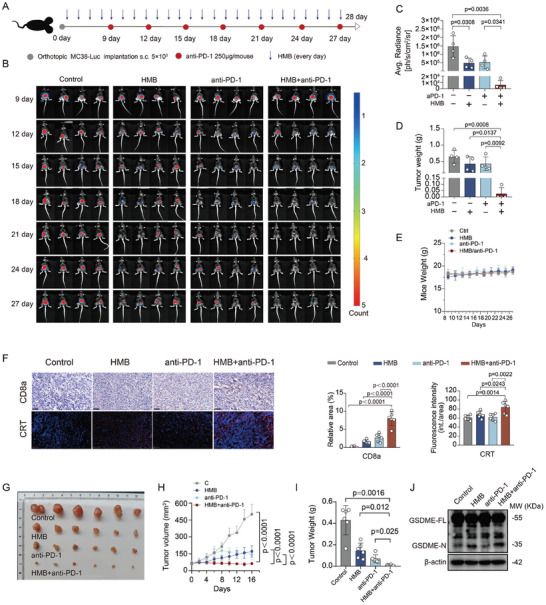
HMB combination enhanced the sensitivity of CRC tumors to anti‐PD‐1 treatment. A) The experimental design involved Orthotopic MC38‐Luc (s.c.) tumor inoculation and treatment schedule in C57BL/6J mice. One day after tumor inoculation, the mice were treated daily with H (10 mg kg⁻^1^), M (60 mg kg⁻^1^), and B (100 mg kg⁻^1^). Nine days after inoculation, the mice also received an intraperitoneal injection of 250 µg/mouse every 3 days. B) Orthotopic MC38‐Luc CRC tumor growth was monitored by evaluating the average radiance within the tumor sites by bioluminescence imaging on days 9, 12, 15, 18, 21, 24, and 27. C) Average radiance of tumor burden (fold changes) calculated through bioluminescence imaging at experimental endpoint/Day 27 from all groups. Data are presented as the means ± S.D (n = 4). D) Tumor weight at experimental endpoint/Day 27 from all groups. Data are presented as the means ± S.D (n = 4). E) Average body weight of mice through therapy. F) IHC staining of CD8a (Blue: nucleus; Brown: expression of CD8a.) and IF staining of CRT (Blue: nucleus; red: expression of CRT) in the orthotopic MC38 tumors with the treatment of normal saline, HMB combination, anti‐PD‐1 or HMB combination + anti‐PD‐1. Scale bars = 50 µm. Data are presented as the means ± S.D (n=6). G) Photographs of the excised tumors from MC38 xenograft‐bearing mice across different treatment groups were captured on day 16 (n = 6). H) Tumor growth curves for all treatment groups, presented as the mean ± S.D (n = 6). I) Tumor weight at the experimental endpoint (day 16) for all groups, presented as the mean ± SD (n = 6). J) The expression of GSDME‐FL and GSDME‐N in the tumor tissues of different treatment groups.

Pyroptosis is an immunogenic form of cell death that promotes antitumor immunity and enhances the therapeutic efficacy of anti‐PD‐1.^[^ ^11^, ^24^ ^]^ We investigated whether the HMB combination could improve the therapeutic effects of anti‐PD‐1 treatment in MC38 orthotopic tumors. For this study, a low‐dose of HMB combination (H 10mg kg⁻^1^, M 60mg kg⁻^1^, B 100mg kg⁻^1^) was administered. C57BL/6J mice with palpable orthotopic tumors were treated with vehicle, HMB alone, anti‐PD‐1 alone, or HMB and anti‐PD‐1 combination (**Figure** **10**A). The combined therapy significantly enhanced anticancer efficacy compared with individual treatments (Figure 10B–D). None of the treated mice experienced weight loss during the study (Figure 10E). H&E staining and blood biochemical analyses revealed no notable toxicity in the heart, liver, spleen, lungs, or kidneys (Figure S9A, Supporting Information). Serum biochemical parameters remained within normal ranges (Figure S9B, Supporting Information), indicating no notable toxicity. Furthermore, the combined therapy significantly increased the expression of immunogenicity markers, such as calreticulin (CRT), and enhanced CD8+ T cell infiltration (Figure 10F). Additionally, the MC38 subcutaneous xenograft model further confirmed the synergistic efficacy of the combined HMB and anti‐PD‐1 therapy (Figure 10G–I). Pyroptosis marker, including GSDME‐N, was also detected in the HMB and combination treatment groups, indicating that HMB‐induced GSDME‐dependent pyroptosis enhanced tumor immunogenicity (Figure 10J). These results suggest that combining HMB with anti‐PD‐1 therapy offers an effective and well‐tolerated therapeutic strategy for mCRC treatment.

## 3 Discussion

Although significant progress has been made in the treatment of mCRC in recent years, therapeutic options remain limited in clinical practice beyond the standard regimens, including FOLFOX and FOLFIRI.^[^ ^4^ ^]^ Additionally, treatment options become increasingly scarce when mCRC becomes refractory to these standard regimens, emphasizing the urgent need to develop new anticancer agents for these patients.

Previous studies have identified the TCM formula Huangqin Houpo decoction as an effective treatment for mCRC. However, the specific active ingredients responsible for its efficacy remain unclear. In this study, we analyzed Huangqin Houpo decoction and its drug‐containing serum using UPLC‐Q‐Orbitrap MS and UPLC‐MS/MS systems to screen for potential anti‐CRC agents. This analysis identified honokiol, nobiletin, baicalin, wogonin, daidzein, and magnolol as having significant inhibitory effects on CRC. Active ingredient combination therapies have garnered increasing attention for their potential synergistic effects. Examples include the combinations of songorine, isoliquiritigenin, and 8‐Gingerol from Sini decoction (SND) to treat dilated cardiomyopathy (DCM),^[^ ^8^ ^]^ geniposide and shanzhiside methyl from the TCM prescription “Yueju” for antidepressant effects,^[^ ^25^ ^]^ and arsenic tetrasulfide, indirubin, and tanshinone IIA from compound Huang Dai tablet can yield enhanced synergy efficacies against acute promyelocytic leukemia (APL),^[^ ^7^ ^]^ etc. Following this approach, we evaluated the synergistic effects between honokiol, nobiletin, baicalin, wogonin, daidzein, and magnolol using the ZIP synergy score. Among these, baicalin, magnolol, and honokiol demonstrated strong synergistic effects. We speculate that the HMB combination may have a synergistic killing effect on CRC. After evaluating various concentrations of these compounds, the optimal combined concentration was ultimately identified. We showed that the HMB combination exerted synergistic anticancer effects in CRC cells and organoids, compared with treatments with single or dual agents.

Our findings further revealed that HMB‐induced cell death was primarily mediated through pyroptosis—a form of programmed inflammatory cell death – rather than other pathways. Hallmark features of pyroptosis, such as cell swelling, plasma membrane blebbing, LDH release, GSDME cleavage, and PI‐positive staining, were observed in HMB‐treated CRC cells. Necroptosis, another cell death pathway, shares some features with pyroptosis, such as cellular swelling, plasma membrane rupture, and LDH release.^[^ ^17^ ^]^ However, the necroptosis inhibitor Nec‐1 did not significantly alter cell viability or LDH release, confirming that HMB‐induced cell death does not proceed via necroptosis. Instead, we identified GSDME, but not GSDMD, as the key executor of pyroptosis in HMB‐treated CRC cells. GSDME knockdown rescued cell death and LDH release, and apoptotic markers confirmed a shift from pyroptosis to apoptosis in GSDME‐knockdown cells. Further investigation revealed that the HMB combination triggered reduced mitochondrial membrane potential, decreased BCL‐2 levels, and increased cytochrome C release, along with elevated expression of cleaved caspase‐9, cleaved caspase‐3, and N‐GSDME proteins. Inhibition of caspase‐3 activity by using caspase‐3 siRNA or the caspase‐3 specific inhibitor Z‐DEVD‐FMK suppressed HMB‐induced pyroptosis, indicating that the mitochondrial intrinsic apoptotic pathway mediated this process. These findings were validated in vivo, where the HMB combination showed significant anticancer effects through caspase‐3/GSDME‐dependent pyroptosis.

Notably, the HMB combination outperformed medium‐ and high‐dose Huangqin Houpo decoction, the FOLFOX regimen, and matched the efficacy of the FOLFIRI regimen in low‐dose applications without notable toxicity. Considering the immunogenicity nature of pyroptosis, the induction of pyroptosis in tumors can convert cold tumors into hot tumors, thereby activating anti‐tumor immunity. So, we explored whether HMB‐induced pyroptosis could enhance anti‐PD‐1 immunotherapy. In combination with PD‐1 blockade, HMB significantly increased the expression of the immunogenic marker CRT and enhanced CD8a+ cell infiltration, thereby augmenting antitumor efficacy in vivo without inducing notable systemic toxicity.

**Figure 11:**
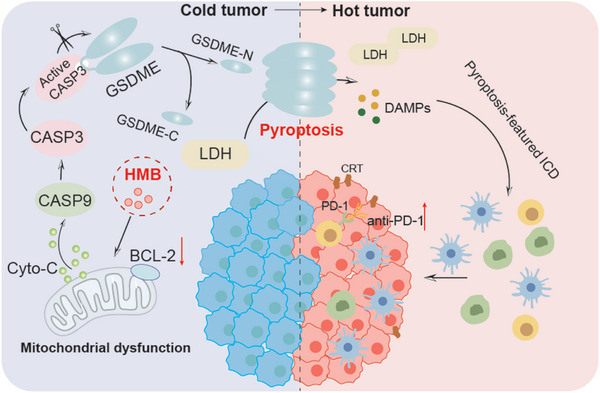
Schematic diagram for the functional mechanism of the HMB combination synergistic anti‐tumor. On entering the cells, HMB induces GSDME‐dependent pyroptosis through the intrinsic mitochondrial apoptotic pathway. Most importantly, the induction of pyroptosis in tumors can convert cold tumors into hot tumors, thereby activating anti‐tumor immunity. When combined with anti‐PD‐1 therapy, the pyroptosis‐triggering HMB combination enhances the therapeutic efficacy of anti‐PD‐1 immunotherapy.

Overall, we identified the HMB combination – honokiol, magnolol, and baicalin – as the principal active components of Huangqin‐Houpu decoction. This combination not only demonstrated significant synergistic anticancer activity, but also enhanced the effectiveness of anti‐PD‐1 immunotherapy, offering a promising therapeutic option for CRC patients (**Figure** **11**). Although the HMB combination has provided promising findings, its clinical application faces several challenges. Optimization of dosage and treatment regimens is crucial, considering individual patient variability. Long‐term safety and potential side effects require further investigation, particularly across diverse cancer patient populations. Moreover, the immunomodulatory effects of the HMB combination and its combination with existing treatments such as chemotherapy, radiotherapy, and targeted therapies, warrant further research. The clinical trials will be crucial for translating these findings into practice. Nonetheless, the HMB combination holds significant anticancer potential for CRC patients.

## 4 Experimental Section

### Preparation of Huangqin Houpo Decoction Formula

According to the 2020 Edition of the Chinese Pharmacopoeia, Huangqin Houpo Decoction, which consists of eight Chinese herbs, was purchased from the Affiliated Hospital of Hangzhou Normal University, Hangzhou, China. All Chinese herbs were identified by a herbal botanist.

### Identification of Compounds and Prototype Compounds in Huangqin Houpo Decoction

The UPLC‐Q‐Orbitrap MS system facilitated the identification of the active ingredients of Huangqin Houpo decoction. First, eight herbs were ground and boiled in 10 times their volume in water for 2 h. After centrifuging at 10,000 rpm for 5 min, the solution of Huangqin‐Houpu decoction was obtained. This solution was then concentrated and dried in a vacuum oven at 60 °C. After drying, 2 mg of the dried substance was weighed and placed in a 1.5 mL centrifuge tube, to which 1 mL of pure water was added, and the mixture was sonicated for 10 min. The supernatant was then centrifuged at 1,4000 rpm for 20 min for analysis. To detect the chemical components in serum, SD rats were treated with the Huangqin Houpo Decoction solution via gavage (4.368 g/dose) for 4 days. Following the last administration, blood samples were collected from the eyelids at the following time points: 5 min, 10 min, 20 min, 40 min, 1 h, 3 h, 6 h, 8 h, and 12 h. Serum metabolome analysis was performed by Wuhan Metwell Biotechnology Co., Ltd.

### Cell Culture

The HCT116 and LoVo cell lines were acquired from the Institute of Basic Medical Sciences, Chinese Academy of Medical Sciences. HCT116 and LoVo cells were cultured in Iscove's Modified Dulbecco Medium (IMDM) and Dulbecco's Modified Eagle Medium/Nutrient Mixture F‐12 (DMEM/F12), respectively, supplemented with 10% FBS. All cells were maintained in a humidified atmosphere with 5% CO_2_ at 37 °C.

### Cell Viability Assay

The cell viability of HCT116 and LoVo cells treated with drugs was determined using the Cell Counting Kit‐8 (Meilunbio, MA0218) assay. The IC_50_ values were calculated using GraphPad Prism 9.0 software (San Diego, CA, USA). The ZIP synergy score was predicted using the online SynergyFinder software (https://synergyfinder.fimm.fi).^[^ ^13^ ^]^ Compusyn software was used to calculate the Combination Index (CI) values.

### Measurement of Cell Death and Cell Cycle

HCT116 and LoVo cells were subjected to the indicated treatments and then were collected. The cell death ratio was measured by flow cytometry using the Phartmingen Annexin V‐FITC Apoptosis Detection Kit I (Becton, Dickinson and Company, 556547). Similarly, the cell cycle was analyzed by flow cytometry using cell cycle staining kit (MultiSciences (Lianke) Biotech, CO., CCS012)

### Colony‐Formation Assays

HCT116 and LoVo cells were plated in 6 cm dishes and the medium was replaced every 3 days. The indicated drug treatment was continued until cell colonies were observed under a microscope. The cells were then fixed and stained with 0.1% crystal violet. Ultimately, the colonies were enumerated using ImageJ.

### Wound Healing Assay

HCT116 and LoVo cells were seeded onto 24‐well plates to form a confluent monolayer, and then a uniform scratch was created using a sterile pipette tip. Subsequently, the cells were treated with specified concentrations of honokiol, magnolol, and baicalin (HCT116: H 4 µg mL⁻^1^, M 24 µg mL⁻^1^, B 45 µg mL⁻^1^; LoVo: H 10 µg mL⁻^1^, M 27 µg mL⁻^1^, B 100 µg mL⁻^1^). Phase‐contrast images of the scratches were captured at 0 and 12 h after the treatment. Image analysis software was used to measure the wound area and calculate the percentage of wound closure in comparison to the initial scratch area. Statistical analysis was performed to assess the significance of the observed differences between the HMB combination group and the control group.

### Construction of Organoids and Ethics Statement

Upon retrieval of the human colon cancer tissues from their storage solution, they were washed multiple times in 5 mL of DPBS. DPBS containing antibiotics was then discarded, and the tissues were finely minced to ≈0.5‐mm^3^ fragments using surgical scissors before placing them into a centrifuge tube containing the tumor tissue‐digestion fluid. The sample was gently shaken at 37 °C on a shaker for 30–60 min. Every 30 min, the digestion was monitored under a microscope to ensure that the cells were being appropriately released. When adequate single cells were obtained in the suspension, any further digestion was stopped by adding 1mL of the serum and 8 mL of DMEM, followed by thorough mixing of the suspension. The cell suspension was then filtered through a 100 µm strainer into a 50 mL centrifuge tube and centrifuged at 4 °C and 2000 rpm for 5 min. If excess red blood cells were present, a red blood cell lysis solution was added, followed by lysis on an ice bath for 2 min. This action is terminated with the addition of 4 mL of 10% FBS DMEM, and re‐centrifugation under the same settings. The supernatant was then discarded and the cells were counted, and then resuspended at a concentration of 1000 cells/µL in organoid culture matrix gel, ensuring that no air bubble was introduced and the sample was placed on ice. These cells were deposited onto a culture plate within matrix gel droplets and incubated at 37 °C for 10 min to solidify without attaching to the plate. After the droplet had solidified, 700 µL of organoid complete medium containing 2% antibiotics was slowly added to envelop the droplet. PBS was also added around the organoid‐seeded wells so as to maintain moisture before placing the plate in an incubator at 37 °C with 5% CO_2_ for further cultivation. This experiment, which constructs Organoids from patient‐derived colorectal cancer samples, has been approved by the Ethics Committee of the First Affiliated Hospital of Zhejiang University, with approval number II T20240182B‐R3.

### RNA Sequencing

Total RNA was extracted by TRIzol (Invitrogen, CA, USA). Illumina HiSeq 4000 sequencing was performed by LC‐BIO Technologies (Hangzhou) Co., LTD.

### Western Blotting

Cells and tumor tissues were harvested and lysed in RIPA buffer containing PMSF (Beyotime, ST506) and phosphatase inhibitors (Apexbio, C5826). Protein concentrations were immediately measured using the BCA Protein Assay Kit (Beyotime, P0012S). The proteins were then denatured using SDS‐polyacrylamide gels (Beyotime, P0015). Subsequently, Western blotting (WB) analysis was performed to detect the protein expressions. The primary antibodies used in this study are as follows: β‐actin (13E5, CST), p‐RB (D20B12, CST), CDK4 (D9G3E, CST), CDK6 (D4S8S, CST), CyclinD1 (E3P5S, CST), GSDMD (ab219800, Abcam), Cleave N‐GSDMD (ab215203, Abcam), GSDME (ab215191, Abcam), PARP (46D11, CST), caspase‐3 (9662, CST), Cleave caspase‐3 (ab2302, Abcam), caspase‐9 (ET1701‐22, HUABIO), BCL‐2 (15071, CST), cytochrome c (E7F8Z, CST), and caspase‐1 (HA722222, HUABIO). The secondary antibodies used in this study are as follows: anti‐mouse IgG (7076, CST), and anti‐rabbit IgG (7074, CST).

### LDH Release Assay

According to the manufacturer's protocol, the LDH release was determined by using an LDH assay kit (Beyotime Biotechnology, C0016) after the indicated treatments.

### siRNAs and shRNA Knockdown

Caspase‐3 in HCT116 and LoVo cells was transiently silenced by transfecting targeted siRNAs using Lipofectamine^TM^ 2000 Transfection Reagent (Thermo Fisher Scientific, 11668019), following the manufacturer's protocol. To establish stable cell lines with a low expression of GSDME, shRNA was employed to silence GSDME in HCT116 and LoVo cells. Stable cell lines exhibiting GSDME knockdown were generated by infecting with retroviruses packaged in HEK293T cells. The viral supernatants were used to infect the target cells for 48 h, followed by selection with puromycin for 7 days to establish stable cell lines. The sequence of all siRNAs and shRNA used in this study is listed in **Table** **1**.

**Table 1:** Sequence of siRNAs and shRNA.

siRNAs or shRNA	Sense (5′‐3′)
Caspase‐3#1:	CCGACAAGCUUGAAUUUAUTT
Caspase‐3#2	UUAUAACUGUUGUCCAGGGTT
Caspase‐3#3	AUAAUAACCAGGUGCUGUGTT
shGSDME	GATGATGGAGTATCTGATCTT

### RT‐PCR

Total cellular RNAs were extracted using TRIzol reagent (Invitrogen, CA, USA), and complementary DNA (cDNA) was synthesized by using the Transcript All‐in‐One First‐Strand cDNA Synthesis SuperMix for RT‐PCR (One‐Step gDNA Removal) (TransGen Biotech, AT341‐03). RT‐PCR was conducted by using the miRNA Universal SYBR qPCR Master Mix (Vazyme, MQ101). The relative expression levels of genes were calculated according to the 2^‐ΔΔCT^ methods. The sequences of qPCR primers are listed in **Table** **2**:

**Table 2:** Primers sequence for Real‐Time qPCR.

Gene	Forward primer	Reverse primer
GSDME	CACACTGTGCCACTTGCTTC	GTCAGCTGAGGCAAACAAGC
β‐actin	ATCATTGCTCCTCCTGAGCG	GTACAAGAAAGTTGGGTAGAAGCA

### JC‐1 Staining Assay

After the specified treatments, the mitochondrial membrane potential was assessed by using the Enhanced Mitochondrial Membrane Potential Assay Kit (Beyotime Biotechnology, C2003S). The measurement was performed via flow cytometry in accordance with the manufacturer's instructions.

### Transmission Electron Microscopy

HCT116 and LoVo cells were seeded to reach 80% confluence after 2 days and then treated with an HMB combination for 24 h. After treatment, the cells were washed with PBS, fixed overnight at 4 °C in 2.5% glutaraldehyde, washed with 4% sucrose in PBS, and stained with 2% osmium tetroxide for 1 h under gentle rotation. Subsequently, the cells were dehydrated through a graded ethanol series (70%, 90%, 95%, 100%) for 10 min at each step, followed by 30 min in propylene oxide. The cells were then incubated in a 1:1 mixture of propylene oxide and resin for 4 h, infiltrated for 2 days, embedded in fresh resin, and polymerized at 70 °C for 24 h. Ultrathin sections (50 nm) were prepared, mounted on mesh copper grids, and imaged using a ThermoScientific Talos L120C transmission electron microscope. Figure 5 E (cell morphology) and Figure 7 A (mitochondrial morphology) are from the same sample.

### In Vivo Assay

1. For the orthotopic colorectal cancer BALB/c nude mouse model, 1 × 10⁶ HCT116‐luc tumor cells were injected into the mice to establish tumors. After 7 days, the mice were randomized into eight groups: control (normal saline containing 1% Tween 80 and 0.1% CMC‐Na), H group (10 mg kg⁻^1^), M group (60 mg kg⁻^1^), B group (100 mg kg⁻^1^), HM group, HB group, MB group, and HMB group. The indicated treatments were administered intraperitoneally every 2 days. Tumor size was monitored using the IVIS Lumina LT imaging system (Biospace PhotonIMAGERTM OPTIMA, 2021248900). The mice were sacrificed after they completed the treatments. Their tissues were collected for HE staining, and blood samples were also collected to evaluate biochemical parameters.
2. For the subcutaneous xenograft BALB/c nude model, 5 × 10⁶ HCT116 cells were injected into the flanks of BALB/c nude mice to establish tumors. Once the tumors reached the appropriate size, the mice were randomly assigned to control (normal saline containing 1% Tween 80 and 0.1% CMC‐Na), H group (10 mg kg⁻^1^), M group (60 mg kg⁻^1^), B group (100 mg kg⁻^1^), HM group, HB group, MB group, and HMB group. Treatments were administered intraperitoneally daily. Tumor volume and body weight were measured every 2 days, with tumor volume calculated as follows: (short diameter)^2^ × (long diameter) / 2.
3. To compare the therapeutic effects of HMB, HQHPD, FOLFOX, and FOLFIRI regimens, 5 × 10⁶ HCT116 cells were injected into the flanks of BALB/c nude mice to construct a subcutaneous xenograft tumor model. Once the tumors achieved an appropriate size, the mice were randomized into six groups: control, honokiol‐magnolol‐baicalin (HMB), Huangqin Houpo decoction‐medium dose (HQHPD‐M), Huangqin Houpo decoction‐high dose (HQHPD‐H), FOLFOX regimen (5‐fluorouracil (5‐FU), oxaliplatin, calcium levofolinate), and FOLFIRI regimen (5‐FU, irinotecan, calcium levofolinate). The FOLFOX group received 5‐FU (22.5 mg kg⁻^1^) and calcium levofolinate (33.75 mg kg⁻^1^) on days 1 and 8, with oxaliplatin (9.375 mg kg⁻^1^) on days 2 and 9. The FOLFIRI group received 5‐FU (22.5 mg kg⁻^1^) and calcium levofolinate (33.75 mg kg⁻^1^) on days 1 and 8, with irinotecan (15 mg kg⁻^1^) on days 2 and 9.^[^ ^26^ ^]^ The HMB group was treated with H (10 mg kg⁻^1^), M (60 mg kg⁻^1^), and B (100 mg kg⁻^1^) daily, while the HQHPD‐M group (The HQHPD‐M dose was converted based on the clinical dosages used for CRC patients) received 15.21 g kg⁻^1^, and the HQHPD‐H group received 30.42 g kg⁻^1^ of the decoction daily. Tumor volume and body weight were measured every 2 days, with tumor volume calculated as follows: (short diameter)^2^ × (long diameter) / 2.
4. For the subcutaneous xenograft C57BL/6J mice model, 5 × 10^5^ MC38 cells were injected into the flanks of C57BL/6J mice to establish tumors. Once the tumors attained an appropriate size, the mice were randomized into 15 groups: control (normal saline containing 1% Tween 80 and 0.1% CMC‐Na), H‐1 group, M‐1 group, B‐1 group, HM‐1 group, HB‐1 group, MB‐1 group, HMB‐1 group, H‐2 group, M‐2 group, B‐2 group, HM‐2 group, HB‐2 group, MB‐2 group, and HMB‐2 group. The indicated treatments were administered intraperitoneally each day (low‐dose (1): H 10 mg kg⁻^1^; M 60 mg kg⁻^1^; B 100 mg kg⁻^1^. Medium‐dose (2):H 15 mg kg⁻^1^; M 90 mg kg⁻^1^; B 150 mg kg⁻^1^). Tumor volume and body weight were measured every 2 days, with tumor volume calculated as follows: (short diameter)^2^ × (long diameter) / 2.
5. For the orthotopic colorectal cancer C57BL/6J mice model, 25 × 10^4^ MC38‐luc tumor cells were injected into the mice to establish tumors. The mice were divided into four groups: control, HMB, anti‐PD‐1, HMB+anti‐PD‐1. The HMB group received H (10 mg kg⁻^1^), M (60 mg kg⁻^1^), and B (100 mg kg⁻^1^) daily (Oral gavage administration), while the anti‐PD‐1 group received 250 µg/mouse every 3 days (Intraperitioneal administration). Tumor size was monitored using the IVIS Lumina LT imaging system (Biospace PhotonIMAGERTM OPTIMA, 2021248900), and the mice were sacrificed after the treatments were completed. Tissue and blood samples were collected for HE staining and for assessing biochemical parameters, respectively.

In all cases, female BALB/c nude mice (age 3–5 weeks) and female C57BL/6J mice (3–5 weeks) were purchased from Shanghai Slac Laboratory Animal Co., Ltd. The Ethics Committee of Scientific Research of Hangzhou Normal University (HSD‐20230829‐05) approved the animal experiments. All treatments were conducted according to the ethical requirements of Ethics Committee of Scientific Research of Hangzhou Normal University.

### Immunohistochemistry Analysis

Tumor sections were deparaffinized, rehydrated, and subjected to antigen retrieval using a pressure cooker in citrate buffer (pH 6.0). Endogenous peroxidase activity was quenched by incubating the sections in 1% H_2_O_2_ for 10 min. The sections were then blocked with 1 × PBS containing 10% goat serum for 30 min and incubated overnight at 4 °C with primary antibodies: anti‐GSDME (Signalway Antibody, #55212‐1, 1:100) and anti‐CD8a (ABclonal, A23305‐PM, 1:50). After the sections were washed, they were incubated with HRP‐conjugated secondary antibodies for 60 min at room temperature. DAB staining was performed to visualize antigen localization, followed by hematoxylin counterstaining. Images were captured using the NanoZoomer S60 Digital Slide Scanner.

### Immunofluorescence Analysis

Tumor sections were deparaffinized, rehydrated, and underwent antigen retrieval in citrate buffer (pH 6.0) using a pressure cooker. Endogenous peroxidase activity was blocked by incubation in 1% H_2_O_2_ for 10 min, followed by permeabilization with 3% Triton X‐100 for 30 min. The sections were blocked with 10% goat serum for 30 min and incubated overnight at 4 °C with the primary antibody CRT (HUABIO, #ET1608‐60, 1:2000). After the sections were washed, they were treated with a fluorescently labeled secondary antibody at room temperature for 60 min. Nuclei were counterstained with DAPI, and images were captured using a Digital Slide Scanner (NanoZoomer S60).

### Statistical Analysis

Statistical analysis was performed using GraphPad Prism 9.0 software. Results are presented as mean ±S.D. The differences between two groups were analyzed by two‐tailed unpaired Student's t‐test, the differences among three or more groups were analyzed by one‐way variance (ANOVA), more groups and factors were analyzed by two‐way variance (ANOVA).

## Conflict of interest

The authors declare no competing interests.

## Author Contributions

X.S., and T.X. designed experiments. Q.S. provided the samples of colorectal cancer patients. Q.G. wrote original manuscript, performed all the experiments. Q.G., Z.Y., Z.Z., L.L., L.X., J.X., Y.Q., J.G., and X.Z. performed the animal studies. G.Q. performed the data statistics analysis.

## Supporting information



Supporting Information

Supporting Information Dataset 1

Supporting Information Dataset 2

## Data Availability

The data that support the findings of this study are available from the corresponding author upon reasonable request.
