# Supplementary material for: Honokiol‐Magnolol‐Baicalin Possesses Synergistic Anticancer Potential and Enhances the Efficacy of Anti‐PD‐1 Immunotherapy in Colorectal Cancer by Triggering GSDME‐Dependent Pyroptosis
Source: Adv Sci (Weinh). 2025 Feb 14;12(13):2417022. doi: 10.1002/advs.202417022 (PMC11967828; doi:10.1002/advs.202417022)
Supplement: Supplementary file 1 — Supporting Information [file ADVS-12-2417022-s003.docx]

**Supplementary Information**

**Honokiol-Magnolol-Baicalin possesses** **synergistic anticancer potential and** **enhances the efficacy of anti-PD-1 immunotherapy in colorectal cancer by triggering GSDME-dependent pyroptosis**

Quan Gao^1, 2, #^, Qinsong Sheng^3, #^, Zijing Yang^1^, ZhiYu Zhu^1^, Lin Li^1^, Lihui Xu^1^, Jing Xia^1^, Yunhao Qiao^1^, Jie Gu^1^, Xiaolong Zhu^1^, Tian Xie^1,^ ^2, *^, Xinbing Sui^1,^ ^2,^ ^*^

^1^ School of Pharmacy, Hangzhou Normal University, Hangzhou, 311121, Zhejiang, China

^2^ State Key Laboratory of Quality Research in Chinese Medicines, Faculty of Chinese Medicine, Macau University of Science and Technology, Macau, P.R. China

^3^ Department of Colorectal Surgery, The First Affiliated Hospital, School of Medicine, Zhejiang University, Hangzhou, Zhejiang, China

^#^ These authors contributed equally to this work.

*Correspondence: [hzzju@hznu.edu.cn](mailto:hzzju@hznu.edu.cn%20) (X.S.) or [xbs@hznu.edu.cn](mailto:xbs@hznu.edu.cn) (T.X.).


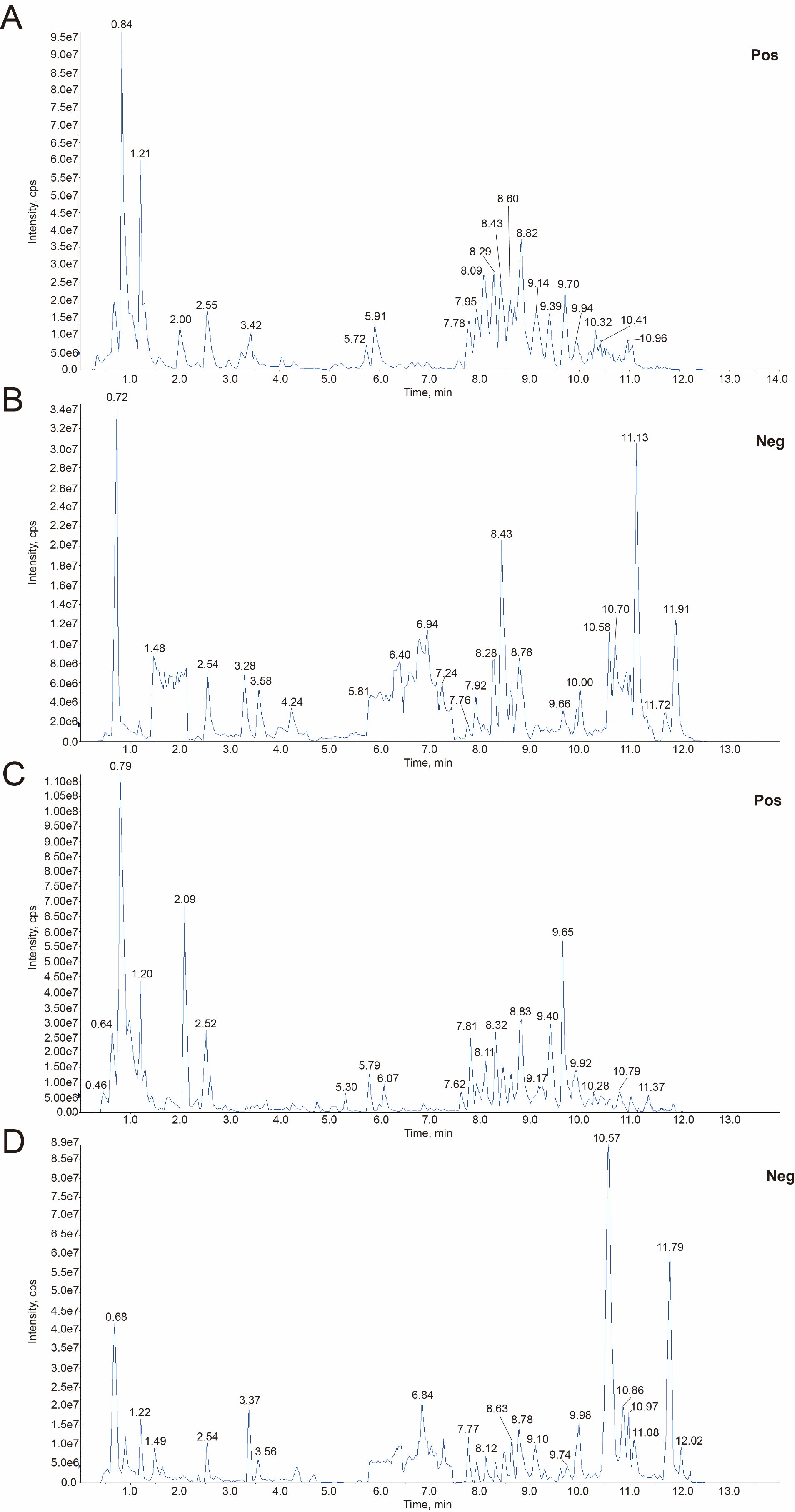


**Figure S1** **The compounds in serum after Huangqin Houpo decoction treatment using UPLC-MS/MS system.** A-B) The chemical base peak ion (BPI) chromatogram in plasma after the treatment of Huangqin Houpo decoction in the negative and positive ion modes. C-D) The BPI chromatogram of blank plasma in the negative and positive ion modes.


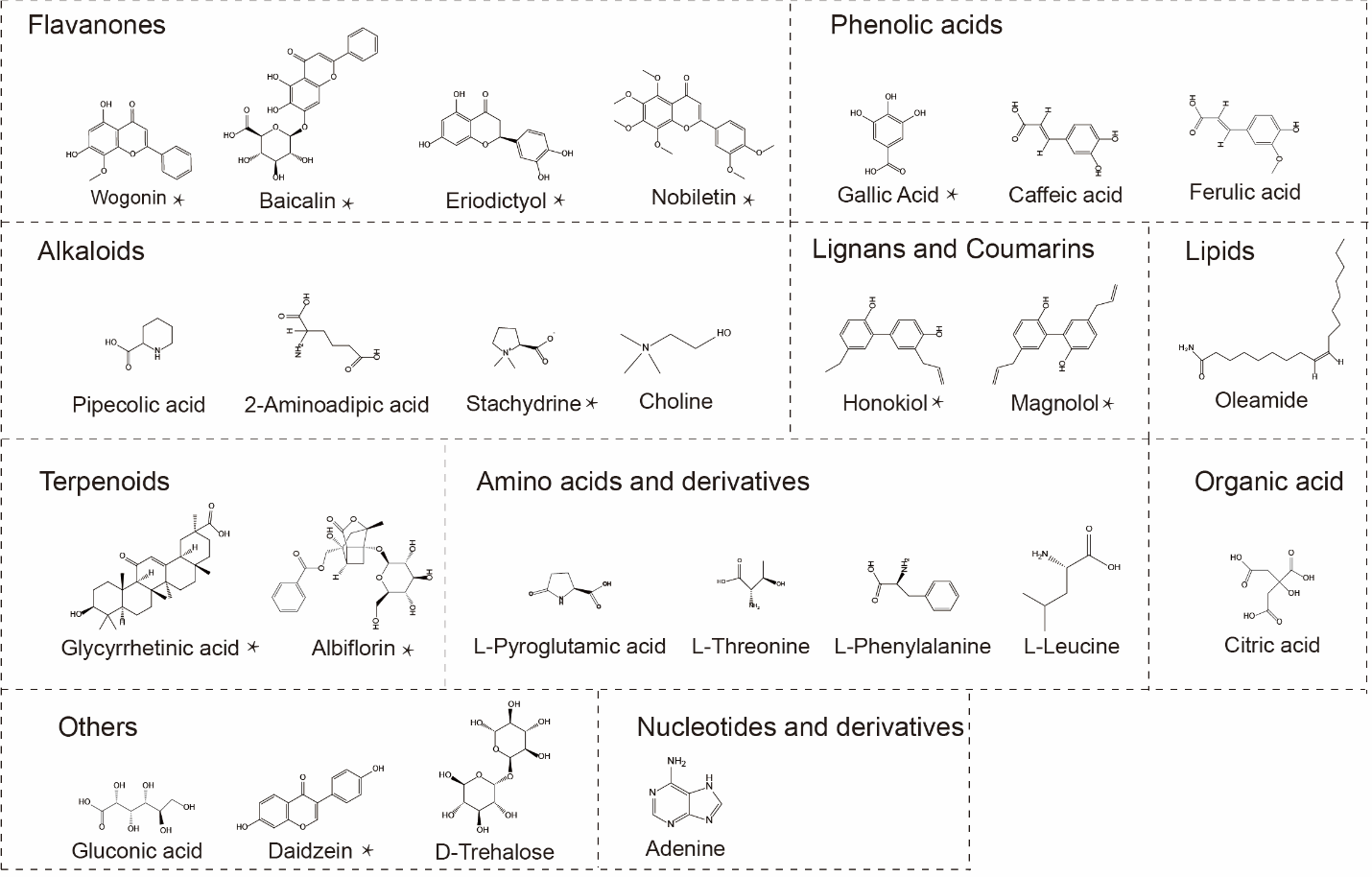


**Figure S2** The structures of the prototype compounds (An asterisk indicates the drugs used for screening).


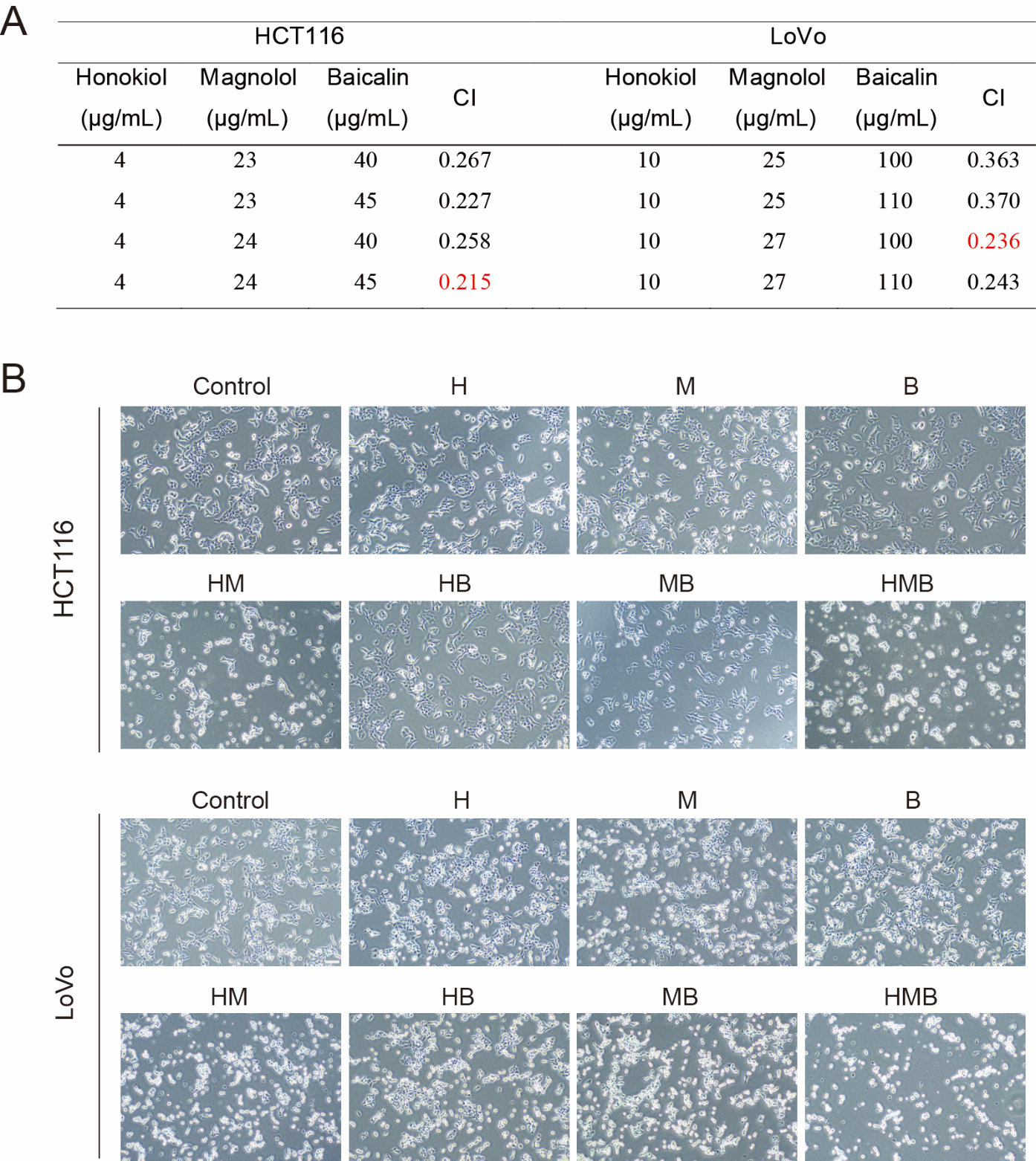


**Figure S3** A) The CI values of HMB combination compatibility were calculated by software Compusyn. B) Cell morphological changes were detected by light microscopy after indicated drug treatment, scale bar = 100 μm.

**
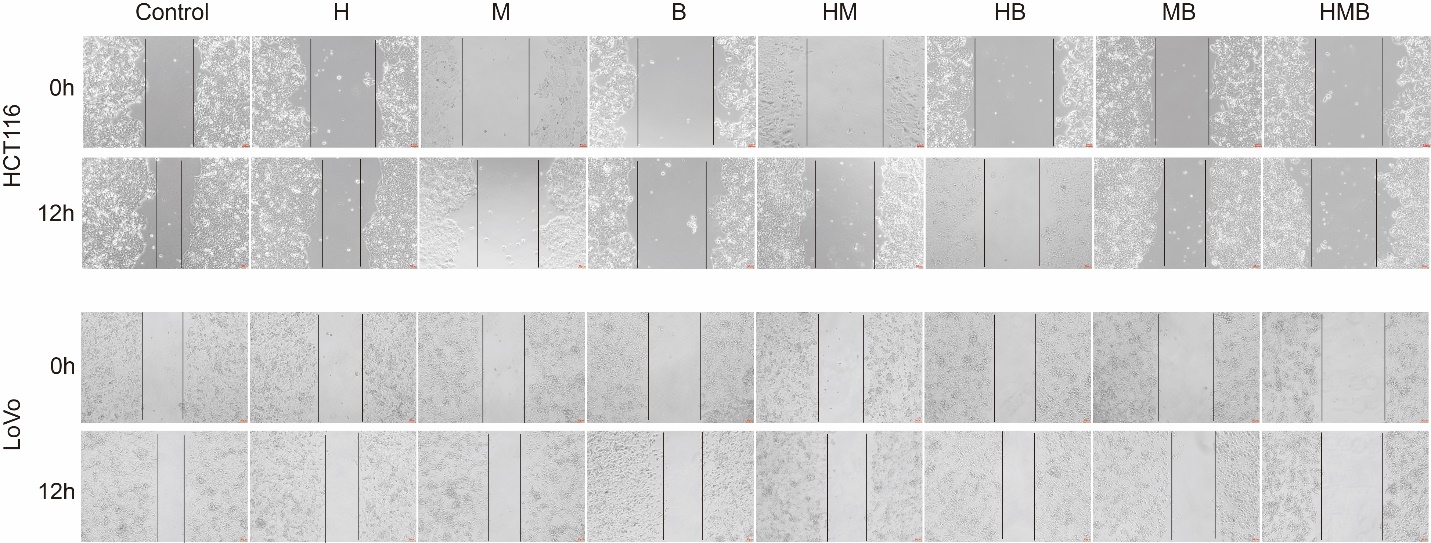
**

**Figure S4** Cell migration was detected by light microscopy after indicated drug treatment. Scale bars: 50 μm.


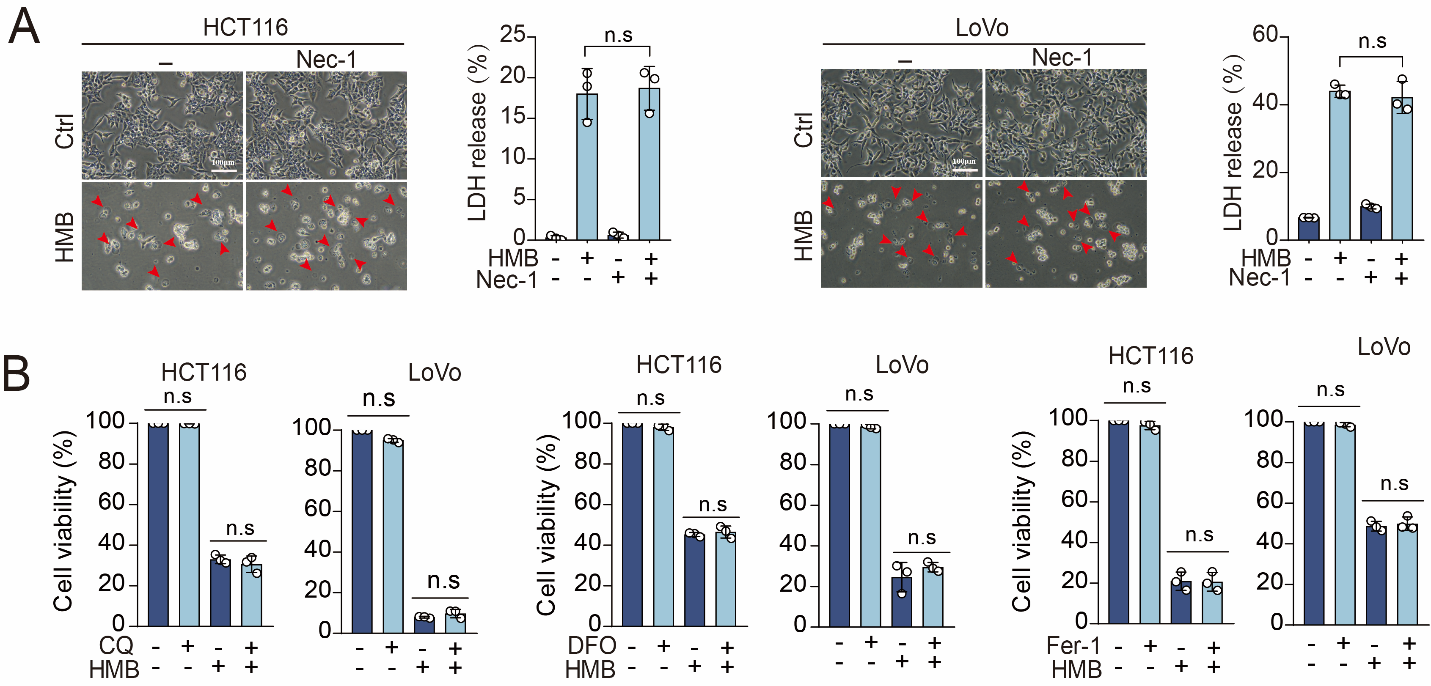


**Figure S5** A) Typical morphological characteristics of pyroptosis were observed after combination treatment of Nec-1 and HMB, including membrane integrity (right) and the LDH release (left), scale bar = 100 μm. B) Cell viability of CRC cells were detected after the treatment with the HMB in combination with different cell death inhibitors for 24h, including the autophagy inhibitors chloroquine (CQ); the ferroptosis inhibitors deferoxamine (DFO) and Ferrostatin-1 (Fer-1).The mean ± SD is shown, n = 3.

**
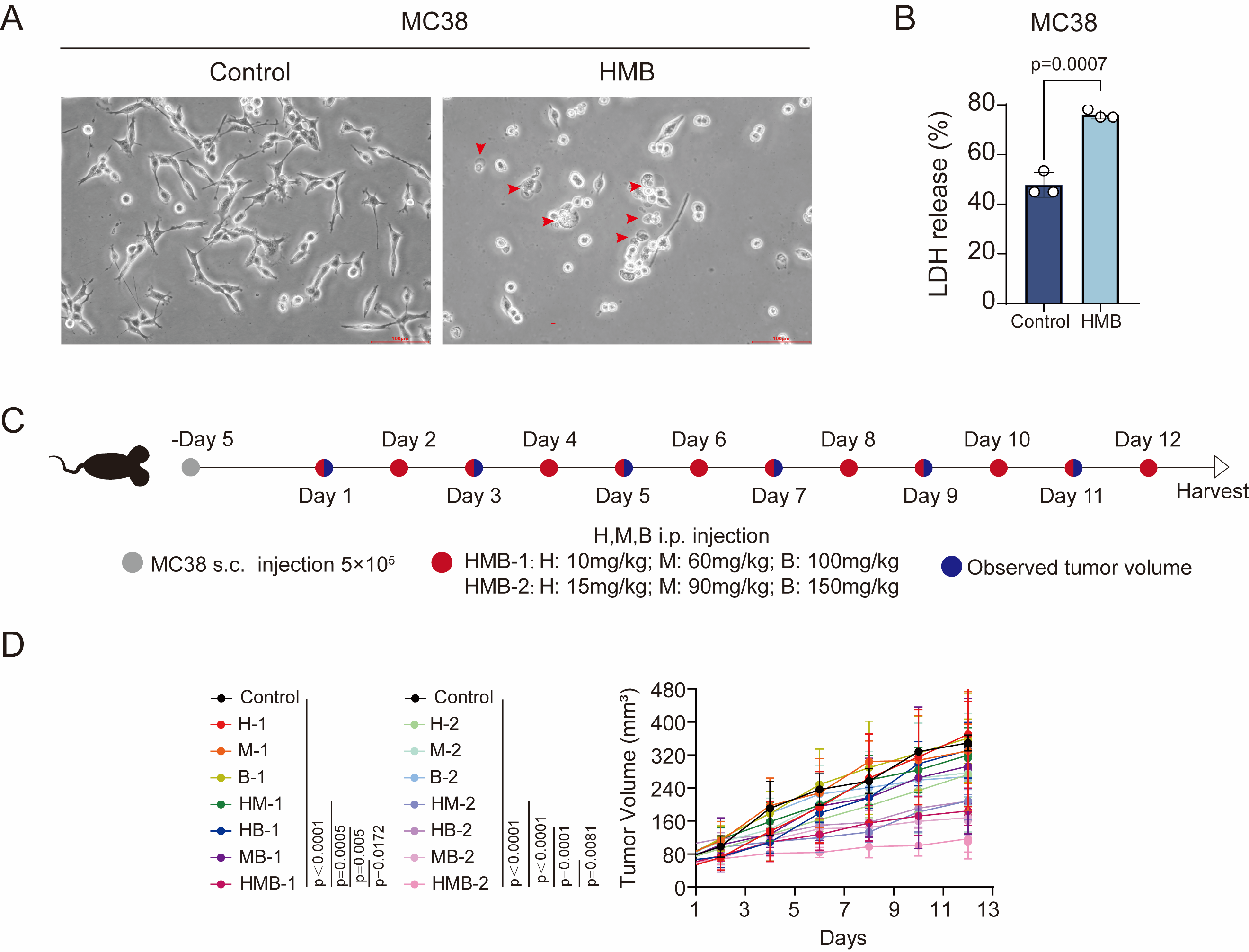
**

**Figure S6** A-B) Typical morphological characteristics of pyroptosis were observed after combination treatment of HMB, including membrane integrity (A) and the LDH release (B), scale bar = 100 μm. C) The experimental design involved subcutaneous (s.c.) tumor inoculation and treatment scheduling in HCT116 tumor-bearing athymic nude mice. Five day after tumor inoculation, mice were treated daily with H (10mg/kg, 15mg/kg), M (60mg/kg, 90mg/kg), and B (100mg/kg, 150mg/kg). D) Tumor growth curve for all treatment groups. Data was shown as mean ± S.D (n = 4).


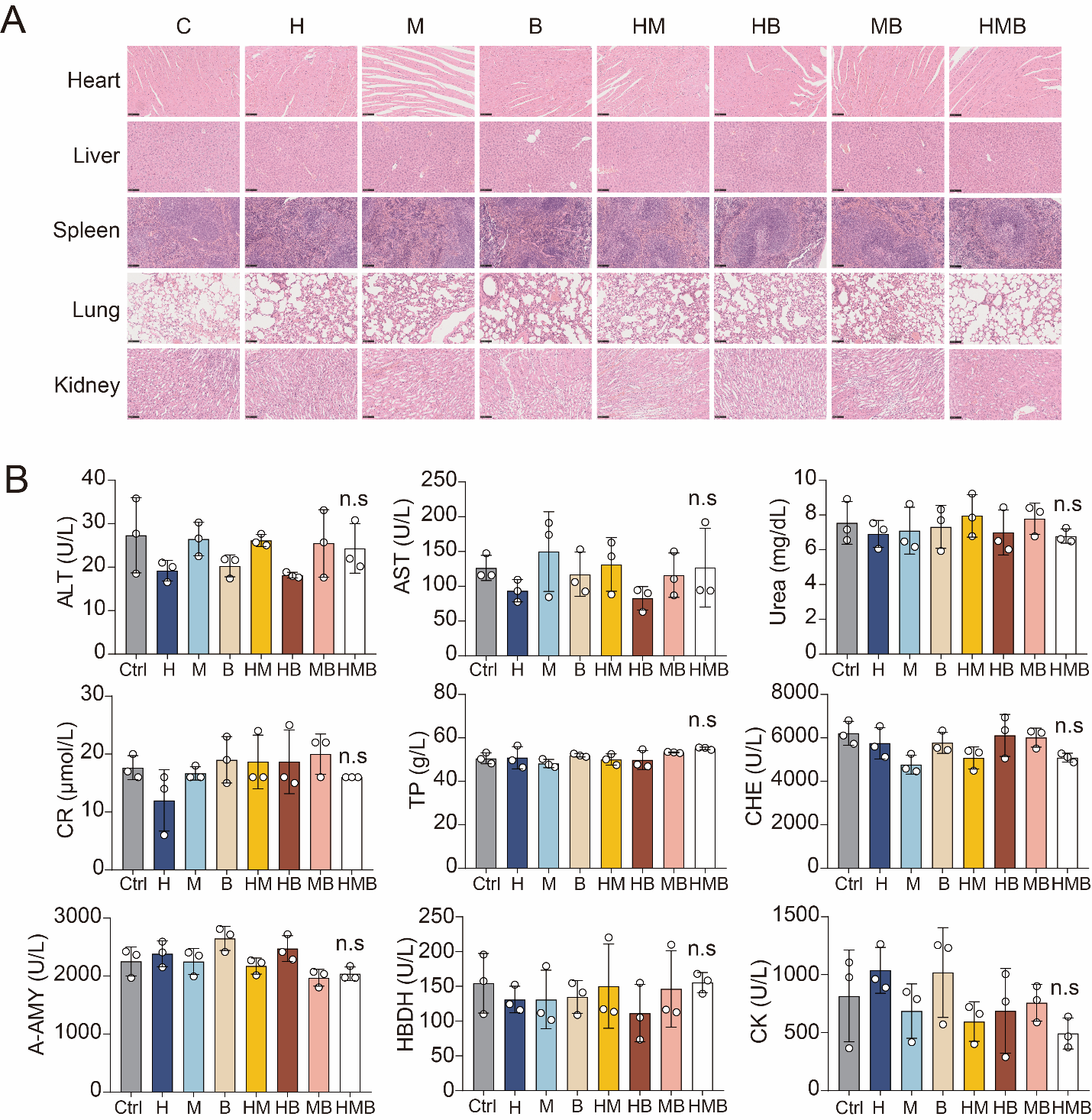


**Figure S7 Safety evaluation of the HMB combination treatment *in vivo*.** A) H&E staining of major organs was analyzed after the drug treatment, scale bar = 100 μm. B) Blood biochemical analysis of important indexes in mice was made.


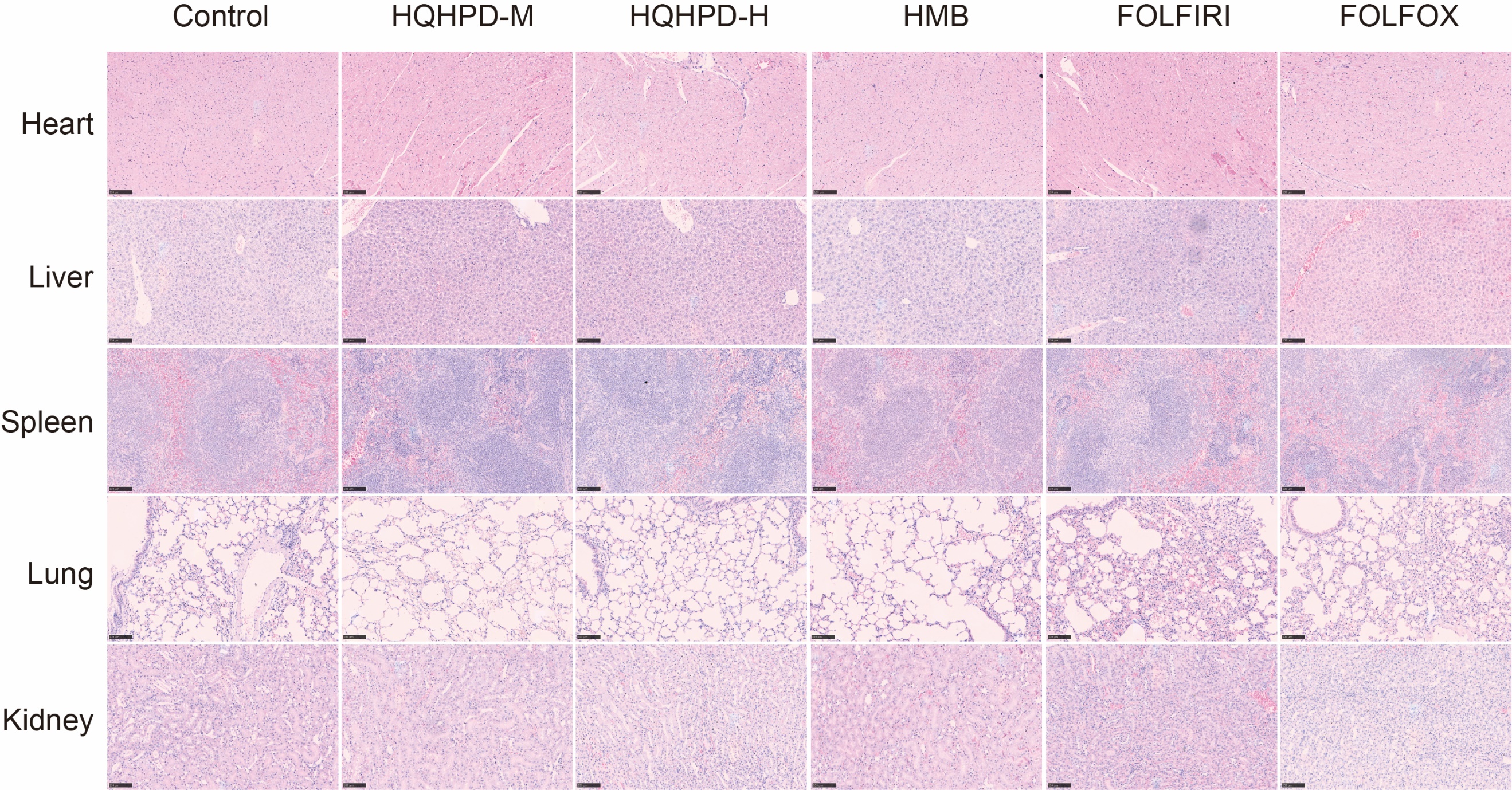


**Figure S8 Comparison between the HMB combination, Huangqin Houpo decoction, FOLFOX and FOLFIRI.** H&E staining of major organs was analyzed after the drug treatment, scale bar = 100 μm.

**
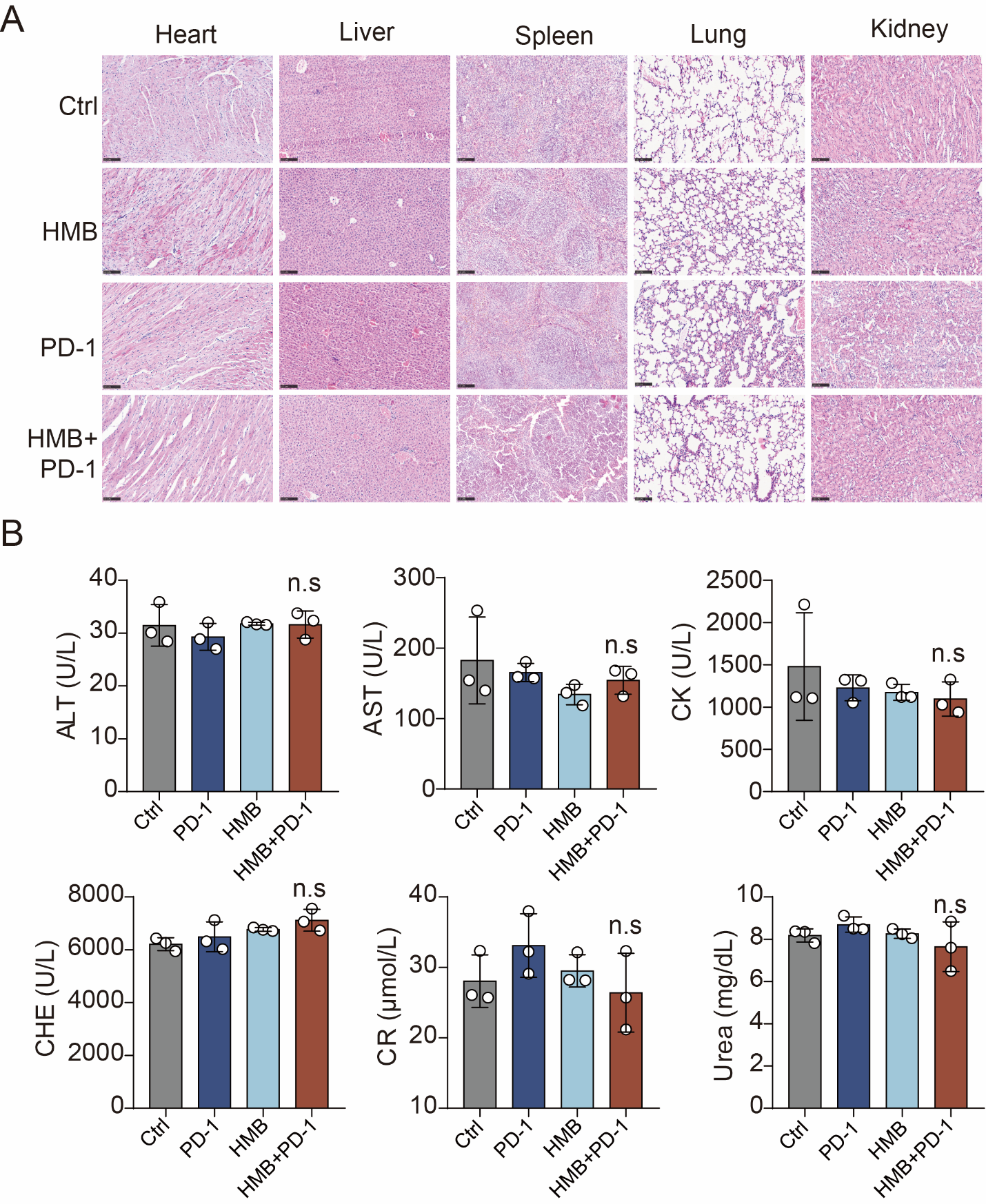
**

**Figure S9 Safety evaluation in orthotopic MC38-Luc CRC tumor model.** A) H&E staining of major organs was analyzed after the drug treatment, scale bar = 100 μm. B) Blood biochemical analysis of important indexes in mice was made.
